# Acute MeCP2 loss in adult mice reveals transcriptional and chromatin changes that precede neurological dysfunction and inform pathogenic cascade

**DOI:** 10.1016/j.neuron.2024.11.006

**Published:** 2024-12-16

**Authors:** Sameer S. Bajikar, Jian Zhou, Ryan O’Hara, Harini P. Tirumala, Mark A. Durham, Alexander J. Trostle, Michelle Dias, Yingyao Shao, Hu Chen, Wei Wang, Hari K. Yalamanchili, Ying-Wooi Wan, Laura A. Banaszynski, Zhandong Liu, Huda Y. Zoghbi

**Affiliations:** 1 Department of Molecular and Human Genetics, Baylor College of Medicine, Houston, TX, 77030, USA; 2 Jan and Dan Duncan Neurological Research Institute at Texas Children’s Hospital, Houston, TX, 77030, USA; 3 Cecil H. and Ida Green Center for Reproductive Biology Sciences, Children’s Medical Center Research Institute, Department of Obstetrics and Gynecology, UT Southwestern Medical Center, Dallas, TX, 75390, USA; 4 Program in Developmental Biology, Baylor College of Medicine, Houston, TX, 77030, USA; 5 Medical Scientist Training Program, Baylor College of Medicine, Houston, TX, 77030, USA; 6 Department of Pediatrics, Baylor College of Medicine, Houston, TX, 77030, USA; 7 Howard Hughes Medical Institute, Baylor College of Medicine, Houston, TX, 77030, USA

## Abstract

Mutations in the X-linked methyl-CpG binding protein 2 *(MECP2)* gene cause Rett syndrome, a severe childhood neurological disorder. MeCP2 is a well-established transcriptional repressor yet upon its loss hundreds of genes are dysregulated in both directions. To understand what drives such dysregulation, we deleted *Mecp2* in adult mice, circumventing developmental contributions and secondary pathogenesis. We performed time-series transcriptional, chromatin, and phenotypic analyses of the hippocampus to determine the immediate consequences of MeCP2 loss and the cascade of pathogenesis. We find that loss of MeCP2 causes immediate and bidirectional progressive dysregulation of the transcriptome. To understand what drives gene down-regulation, we profiled genome-wide histone modifications and found that a decrease in histone H3 acetylation at down-regulated genes is amongst the earliest molecular changes occurring well before any measurable deficiencies in electrophysiology and neurological function. These data reveal a molecular cascade that drives disease independent of any developmental contributions or secondary pathogenesis.

## eTOC BLURB

Bajikar et al. delete *Mecp2* in adult mice to assess if molecular dysfunction precedes neurologic dysfunction related to Rett syndrome, independent of development or secondary pathogenesis. They found that MeCP2 acutely regulates the transcriptome and chromatin landscape, and that molecular dysregulation preceded circuit-level or neurological dysfunction.

## Graphical Abstract

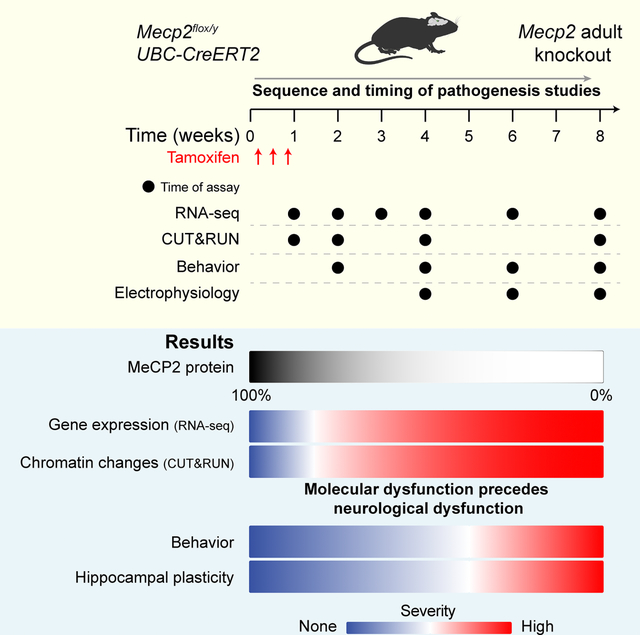

## INTRODUCTION

Loss-of-function mutations in the X-linked gene methyl-CpG binding protein 2 (*MECP2*) cause Rett syndrome (RTT; OMIM: #312750)^1^. RTT, one of the most prevalent childhood neurological disorders, is characterized by a 6–18-month period of apparently normal development followed by regression and a progressive loss of language and motor skills. RTT patients also experience stereotyped hand movements, anxiety, intellectual disability, and seizures^2^. Despite identifying loss-of-function mutations in *MECP2* as the cause of RTT, the precise molecular mechanisms by which loss of MeCP2 function drives pathogenesis remain unknown.

*MECP2* encodes a methyl-cytosine di- and tri-nucleotide binding protein that is most highly expressed in neurons^3,4^. MeCP2 binds throughout the genome to finely tune gene expression. Loss of MeCP2 function causes the dysregulation of hundreds of genes and results in the de-repression of repeat elements such as LINE-1 (L1) retrotransposable elements in the brain^4–8^. Loss of MeCP2 function also causes changes to the chromatin landscape, both locally near genes and globally in localization within the nucleus, further implicating the role of MeCP2 as a fine tuner of global gene expression^3,9–13^. Importantly, MeCP2 has a demonstrated function as a transcriptional repressor and interacts with co-repressor complexes like the NCoR/SMRT complex, yet genes are both up- and down-regulated upon abnormal changes in MeCP2 protein levels or function^5,14–16^. To reconcile these perplexing data, several hypotheses have been set forth.

First, widespread transcriptional dysregulation is not observed in neonatal MeCP2-deficient brains^9,17^, which has led to the hypothesis that these broad transcriptional changes are a consequence of a post-natal developmental role of MeCP2, as MeCP2 expression increases during post-natal brain development^3,18^. This post-natal upregulation is concomitant with the post-natal deposition of non-CpG methylation in the brain, enriched in genes dysregulated by loss of MeCP2^19–23^.

Second, conclusions regarding the molecular function of MeCP2 have been typically drawn from transcriptional profiles generated in symptomatic mouse models of RTT. Symptomatic RTT brains exhibit reduced neuronal size, reduced dendritic arbors, and disrupted synaptic and circuit activity^24–27^. These severe phenotypes have led to an alternative hypothesis that the broad transcriptional changes, particularly gene down-regulation, could be a secondary consequence of reduced neuronal function and health, rather than primary to MeCP2 molecular function.

Countering the first consideration, proper MeCP2 function is required throughout life. Conditional deletion of *Mecp2* in adult mice reproduces all phenotypic deficits and premature lethality of the *Mecp2* germline-knockout animal^28–31^. These data suggest that a potential early post-natal developmental role for MeCP2 does not account for the broad transcriptional changes; however, this has not been experimentally tested. Such an analysis will provide an opportunity to identify gene expression changes that are most proximal to MeCP2 loss and distinct from changes secondary to neuronal and behavioral dysfunction.

To address these gaps in knowledge, we conditionally deleted *Mecp2* in adult mice and systematically performed transcriptomic, epigenomic, and phenotypic assessments at multiple time points after loss of MeCP2. We found that adult deletion of *Mecp2* causes early transcriptional changes that became more robust over time and mirrored those of the *Mecp2* germline null mice. Interestingly, genes persistently dysregulated upon loss of MeCP2 are both up- and down-regulated, highly methylated at non-CpG sites (mCH), and lose MeCP2 occupancy at a faster rate than non-persistently dysregulated genes. Furthermore, persistently dysregulated genes exhibit an early corresponding local change in H3K9ac and H3K27ac, suggesting that the chromatin state is reflective of the gene expression changes proximal to MeCP2 function. Lastly, the adult MeCP2 knockout mice display circuit-level electrophysiologic deficits weeks after the dysregulation of the transcriptome. Altogether, our data demonstrate that the bidirectional gene expression changes that occur upon loss of MeCP2 are independent of development or secondary pathogenesis and establish the timing and cascade of events upon loss of MeCP2.

## RESULTS

### Acute genetic deletion of Mecp2 in adult mice

**Figure 1.**
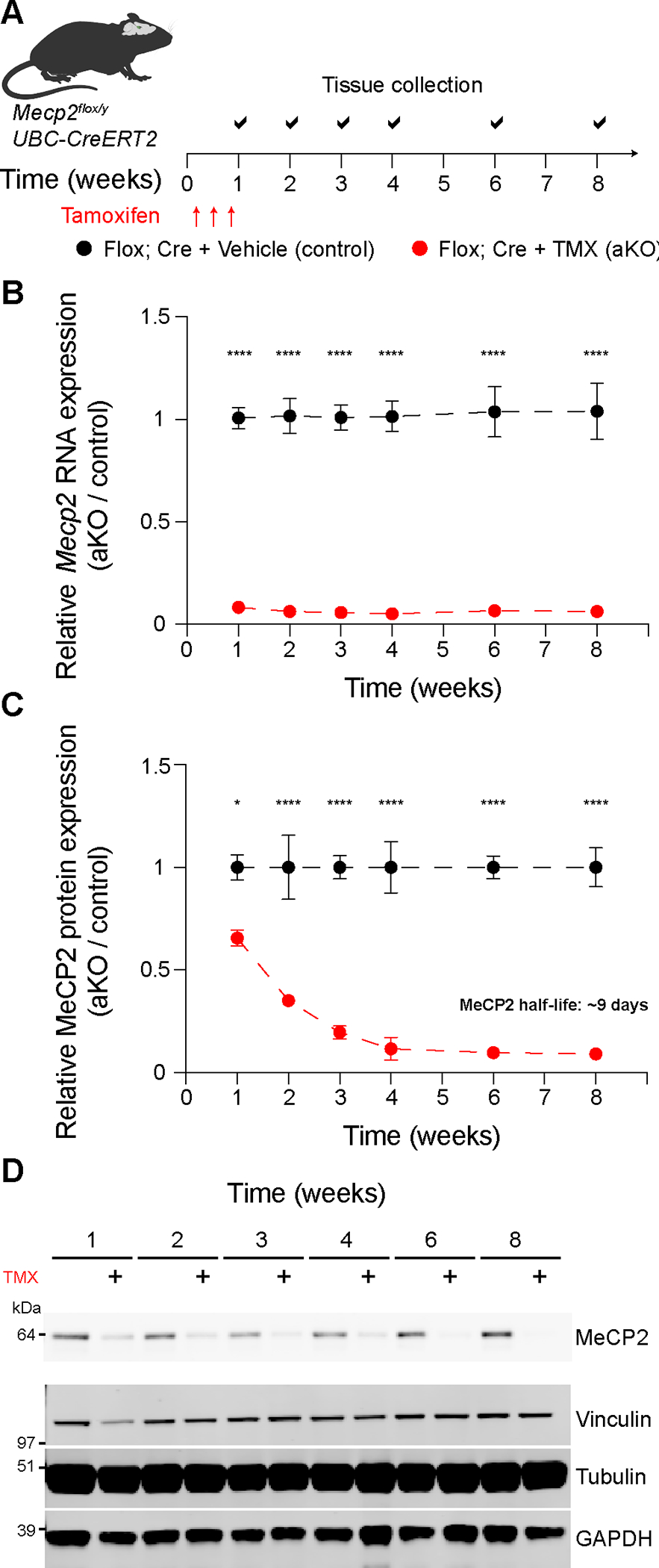
Acute depletion of MeCP2 protein in adult knockout mice. (A) Schematic of conditional deletion of *Mecp2* in 16-week old, adult *Mecp2*^flox/y^; *UBC-CreERT2* mice using tamoxifen (TMX). Male mice were treated with 3x doses of either TMX (100 mg/kg) or peanut oil vehicle. TMX-treated animals are referred to as adult knockout (aKO) and shown in red, while vehicle-treated animals are referred to as control and shown in black. (B) Quantification of *Mecp2* RNA expression in the hippocampus after TMX treatment. Data are normalized to control *Mecp2* expression at the Week 1 timepoint. (C) Quantification of MeCP2 protein expression in the hippocampus after TMX treatment by western blot. MeCP2 protein band intensity was normalized to a panel of loading controls, and each timepoint was normalized to the average of the control MeCP2 quantification per time point. (D) Representative western blot image of MeCP2 and loading controls per time point. Data displayed as mean ± sem from *n* = 2–4 biological replicates. Data were analyzed by two-way ANOVA and post-hoc multiple comparisons, with (*) *P* < 0.05 and (****) *P* < 0.0001.

To circumvent any potential contributions of development or secondary pathogenesis such as reduced neuronal function on MeCP2-dependent gene expression, we sought to acutely delete *Mecp2* in adult male mice starting at sixteen weeks of age, ten weeks after MeCP2 levels have stabilized, DNA methylation has been deposited, and the brain is acutely dependent on MeCP2 function^18,19,29,31,32^. We bred a floxed *Mecp2* allele (Flox; *Mecp2*^*tm1.1Jae*^;^26^) with a ubiquitously expressed CreER recombinase (Cre; *UBC-CreERT2*) to generate Flox; Cre mice and associated control littermates. We used only male mice as this study is focused on the molecular effects of MeCP2 without the confounds of random X inactivation. In this study, we will refer to tamoxifen-treated Flox; Cre mice as *Mecp2* adult knockout mice (aKO) and vehicle-treated Flox; Cre mice as control. Additionally, tamoxifen-treated Flox-only, Cre-only, and wild-type littermate mice will be referred to as treatment controls. Using a one-week, three-dose regimen of 100 mg/kg tamoxifen delivered intraperitoneally (Figure 1A), we found that *Mecp2* RNA is reduced by over 90% within one week in the aKO hippocampus, a brain region dependent on precise MeCP2 protein levels^18,28,33–35^ (Figure 1B). These data demonstrate a short pulse of tamoxifen robustly recombines the *Mecp2* genomic locus in the brain.

We next sought to determine the dynamics of *Mecp2* RNA and protein loss in the aKO brain. We first found that *Mecp2* expression was largely unchanged and stable over eight weeks in all control and treatment control groups (Figure S1A). Additionally, MeCP2 protein levels were stable in control hippocampi over the course of eight weeks (Figure S1B,C). In aKO mice, MeCP2 protein is significantly (*p* < 0.05) reduced by ~30% within one week, a decrease in MeCP2 protein level that is sufficient to cause neurological dysfunction^18^. Furthermore, MeCP2 protein continues to exponentially decrease and was reduced by over 90% four weeks after treatment. Using these data, we quantitatively estimated that hippocampal MeCP2 protein has a half-life of ~9 days *in vivo* (Figure 1C,D). These data demonstrate that MeCP2 protein levels are stable in adult mice and that conditional genetic deletion of *Mecp2* in adult mice acutely reduces MeCP2 protein to a pathogenic level by one week.

### Progressive transcriptional dysregulation occurs upon loss of MeCP2 independent of development

**Figure 2.**
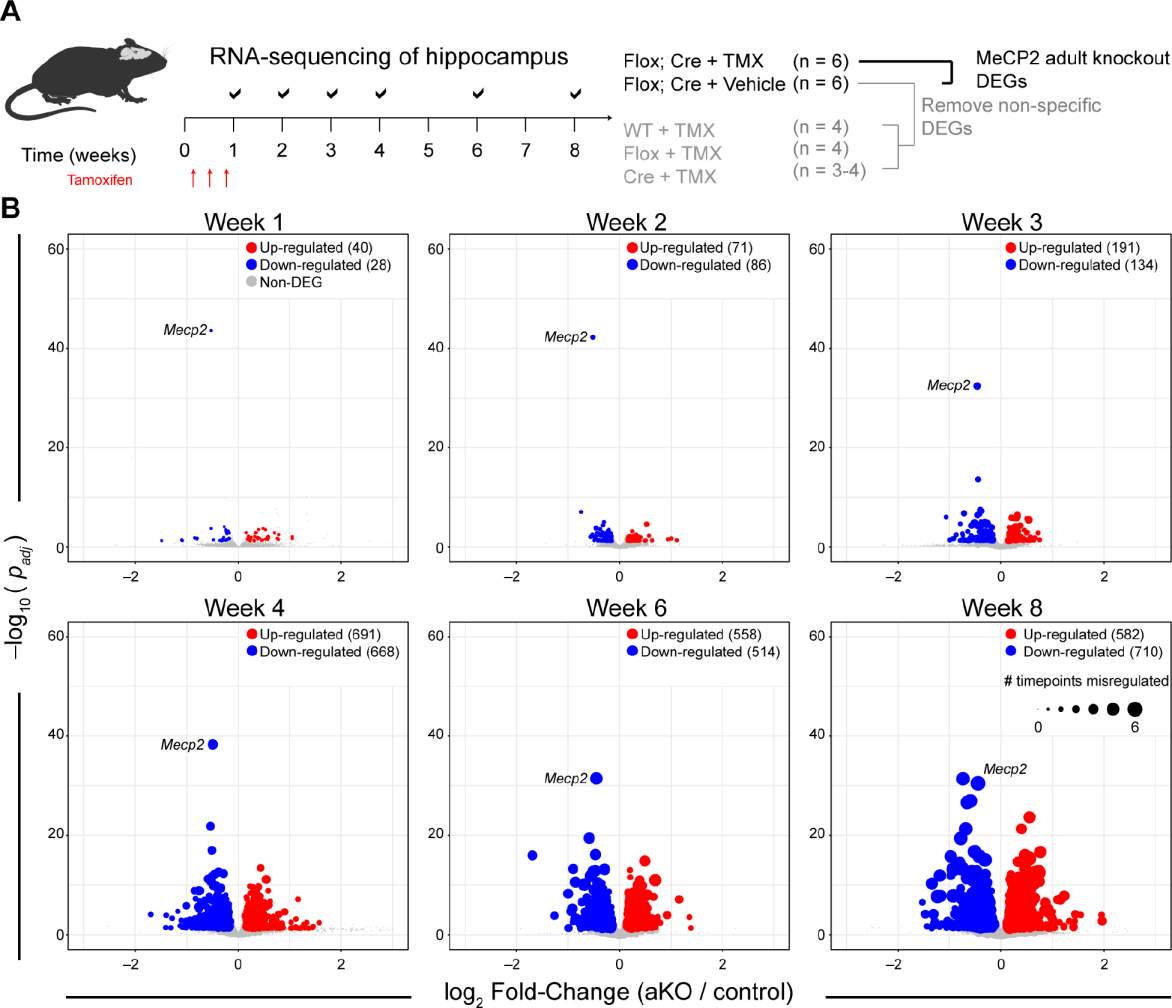
Progressive global transcriptional dysregulation in the hippocampus after acute depletion of MeCP2. (A) Schematic of conditionally deleting *Mecp2* in 16-week-old, adult *Mecp2*^flox/y^; *UBC-CreERT2* mice using tamoxifen (TMX). Male mice were treated with 3x doses of either TMX (100 mg/kg) or peanut oil vehicle. Total RNA from the hippocampus was collected at the time points indicated by a check mark after TMX treatment in adult knockout (aKO), control, and treatment control mice. Bulk mRNA-sequencing was performed and differentially expressed genes (DEGs) between 1) treatment controls and control (Table S2) and 2) aKO and controls were tabluated (Table S4). DEGs detected in treatment controls were removed as non-specific gene expression changes. (B) Volcano plots plotting log_2_ Fold-Change (aKO / control) versus *p*_*adj*_ highlighting differentially expressed genes (DEGs) at each time point. The size of the data points (see right upper center of Week 8 graph) reflects the number of total timepoints a given gene was classified a DEG (*p*_*adj*_ < 0.05 and | log_2_ Fold-Change | > 0.15).

The rapid depletion of MeCP2 allows us to identify the molecular events that are most proximal to reduced MeCP2 levels and function. To globally assess the molecular state upon acute depletion of MeCP2, we performed bulk RNA-sequencing from whole hippocampi at multiple time points in aKO, control, and treatment control male mice starting at one week after tamoxifen treatment (Figure 2A). We collected weekly samples for the first four weeks and at six- and eight-weeks after tamoxifen treatment (Figure 1C). We collected six replicates per time point for aKO and control conditions, and three to four replicates per time point for treatment control conditions. We then deeply sequenced the libraries (~80+ million paired-end reads per sample) to enable the identification of the small-magnitude gene expression changes observed in MeCP2-mutant transcriptomes^5,6,9,20,21^ (Table S1). Consistent with our qPCR results, *Mecp2* RNA was significantly reduced (*p*_*adj*_ *<* 1×10^−43^) within one week of tamoxifen treatment in aKO samples, while unchanged in control and treatment control samples over the course of eight weeks (Figure S2A). We also identified genes that were modulated by acute tamoxifen treatment, like *Irf7*, (Figure S2B) or acute nuclear CreER localization and activity, like *Ackr2* (Figure S2C and Table S2). Identification and removal of these non-specific transcriptional changes allowed us to focus on genes acutely and specifically regulated by MeCP2.

We observed that the MeCP2-dependent transcriptional changes occur as early as one week, when MeCP2 is reduced by ~30%, where we detected 68 significantly differentially expressed genes (DEGs). Of these genes, 40 genes were up-regulated and 28 genes down-regulated. The number of genes dysregulated increased from one- to four-weeks, after which we identified over a thousand genes altered at the four-, six-, and eight-week time points (Figure 2B and Table S2). This global dysregulation of gene expression results in a progressive separation of aKO and control conditions over time (Figure S2D). Additionally, we observed no separation amongst the control samples with respect to time point, suggesting the identified DEGs are specific to reduced MeCP2 levels (Figure S2D). We did not observe a bias in the direction of change in the DEGs, with a nearly half of the DEGs at any given time point being up- or down-regulated (Figure 2B). We also did not observe a relationship between DEG direction and expression level (Figure S2E). Furthermore, we identified genes that were repeatedly dysregulated at multiple time points, with ~30% of the genes dysregulated at one week also dysregulated at eight weeks. The DEG list of each time point significantly (*p* < 0.05) overlaps with every other time point, identifying a consistent set of genes dysregulated by MeCP2. Of the genes dysregulated at eight weeks, ~60% were altered in at least one additional previous time point (Figure S2F). Taken together, our data demonstrate that loss of MeCP2 in adulthood triggers global and progressive dysregulation of the transcriptome independent of development and secondary pathogenesis.

Given our bulk RNA measurements in the hippocampus included a mixture of cell types, we next asked if the observed DEGs were attributed to any specific cell types. We used CIBERSORTx to deconvolute the bulk profiles into a set of single-cell type profiles to estimate the contribution of cell types in the bulk RNA-seq samples collected (see Methods). We found that over 50% of the bulk gene expression comes from excitatory neurons, while inhibitory neurons comprised approximately 5% of the cells in the expression profile. Microglia, oligodendrocytes, and astrocytes comprised approximately 10–15% each towards the expression profile, while oligodendrocyte precursors comprised less than 1%. Importantly, adult deletion of *Mecp2* did not significantly alter the proportion of cell types in the overall expression profile (Figure S3A).

Next, we next asked if the DEGs upon loss of MeCP2 per time point were most attributed to any cell type. At the terminal eight-week time point, excitatory neurons contributed the greatest towards the DEGs (49%), inhibitory neurons contributed the second most (15%), and the glial cell types contributed less than 13% each. The cell-type specific contributions are less prominent at earlier time points, but in almost all cases, excitatory neurons contribute the most. The exception is at the one-week time point, where microglia and astrocytes have a nearly equal contribution (Figure S3B). To further investigate the consequences of this cellular heterogeneity, we performed gene ontology analysis on the bulk differential gene expression (Figure S4 and Table S3). We found terms related to immune function were enriched from the up-regulated DEGs at the one-week and two-week time points, potentially reflecting the glial cell-type contribution identified from deconvolution. At the later six- and eight-week time points, the upregulated DEGs were associated with developmental and neuronal processes. For the down-regulated DEGs, the early one-week time point featured terms related to metabolic processes, while from the two-week time point onward, the DEGs were primarily associated with cell signaling and neurodevelopment.

Overall, our data indicate that the DEGs observed in our bulk RNA-seq measurements largely reflect changes in neurons, particularly excitatory neurons. These analyses suggest that an early neuronal, metabolic, and immune response program is initiated following the loss of MeCP2. From two weeks onward, this response becomes dominated by alterations in neuronal function.

### Transcriptional dysregulation caused by loss of MeCP2 in adulthood is similar to the transcriptional dysregulation caused by germline loss of MeCP2

**Figure 3.**
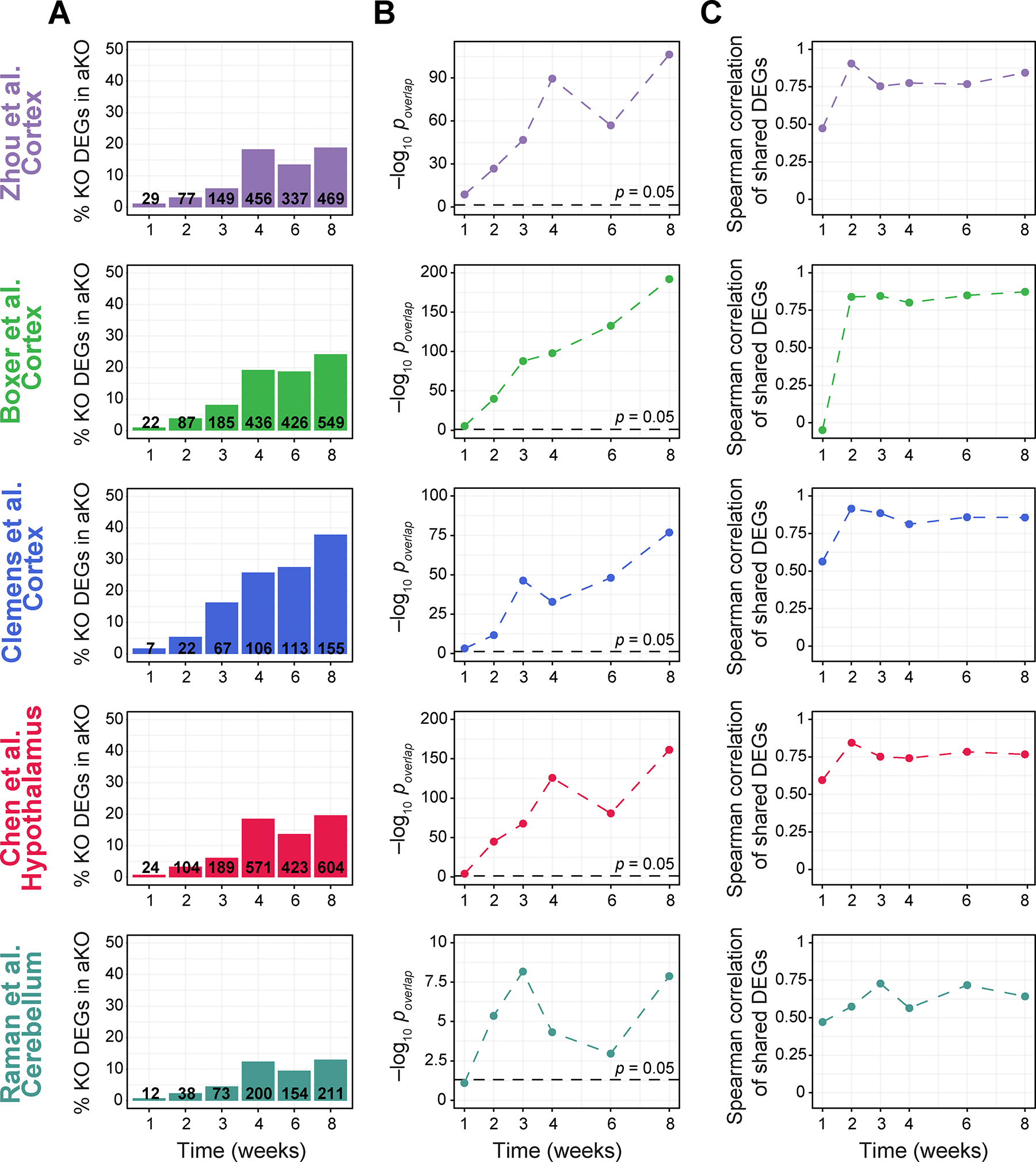
Transcriptional dysregulation caused by loss of MeCP2 in adulthood is similar to the transcriptional dysregulation caused by germline loss of MeCP2. Differential gene expression (DEG) from aKO mice (Figure 2) were compared to five previously published *Mecp2* germline null RNA-sequencing studies from various brain regions, labeled by text to the left of each row. For each *Mecp2* germline null RNA-sequencing study, the number and percentage of aKO DEGs present in the germline, the probability of overlap, and the Spearman correlation between shared DEGs was calculated for each timepoint of aKO samples collected. (A) The percentage of germline DEGs that overlap between the respective *Mecp2* germline null study and aKO. The total number of DEGs shared between each comparison is shown in text on the respective bar. (B) The overlap significance between the aKO and *Mecp2* germline null studies over time. The probability of overlap was computed by Fisher’s exact test. Dashed line indicates *p* = 0.05. (C) Spearman correlation coefficient of the log_2_ Fold-Change (aKO / control) and log_2_ Fold-Change (*Mecp2* germline null / wild-type control) of shared DEGs per time point.

The global, yet subtle, gene dysregulation at eight-weeks after depletion of MeCP2 is reminiscent of the transcriptional dysregulation observed in *Mecp2* germline null brain tissues^5,6,9,10,20,21,36^. We compared the DEGs and directionality of transcriptional changes in the aKO model with previously published transcriptional profiles generated in *Mecp2* germline null mice from multiple brain regions and labs^9,10,20,36,37^. For each data set, we computed the number of shared DEGs, the probability of DEG overlap, and the Spearman correlation coefficient of the log_2_ fold-change amongst the shared DEGs. Firstly, we observed that the percentage of *Mecp2* germline null DEGs observed in the aKO model increases over time, suggesting the aKO transcriptome becomes more similar to *Mecp2*-germline null over time (Figure 3A). Secondly, the extent of DEG overlap was significant at each time point between aKO DEGs and *Mecp2* germline null DEGs from the cortex and hypothalamus. In the cerebellum, the overlap was significant after the first week time point (Figure 3B). Lastly, the Spearman correlation between the log_2_ fold-changes of shared DEGs was over ~0.75 for all the time points after one week in the cortex and hypothalamus, while the correlation was more modest (~0.6) with the cerebellum germline-knockout DEGs (Figure 3C). This suggests that the direction of gene expression alterations that are shared between the aKO and *Mecp2* germline null transcriptome is largely concordant.

Single amino acid substitutions, rather than full gene deletion, in *MECP2* comprise the most common RTT-causing mutations. Therefore, we also compared the aKO transcriptome to transcriptomes generated from mouse models harboring a methyl-cytosine binding domain (MBD) mutation, *Mecp2*-G118E^38^ (Figure S5A), or harboring a transcriptional repression domain (TRD) mutation, *Mecp2*-R306C^9^ (Figure S5B). In the transcriptomes of both mutant models, we observed significant overlap in DEGs across all time points. We also observed the number of shared DEGs increasing over time after loss of MeCP2. Furthermore, the shared DEGs showed a high Spearman correlation over ~0.75 for all time points except for the first week time point (Figure S5). These data suggest that the aKO transcriptome reflects molecular changes observed upon disruption of the functional domains of MeCP2. Taken all together, these data suggest that MeCP2 regulates a core set of transcripts independent of development and brain region, and that the transcriptional cascade induced upon loss of MeCP2 in adult mice converges upon a *Mecp2* mutant-like molecular state.

### Loss of MeCP2 triggers persistent dysregulation in a responsive subset of highly methylated genes

**Figure 4.**
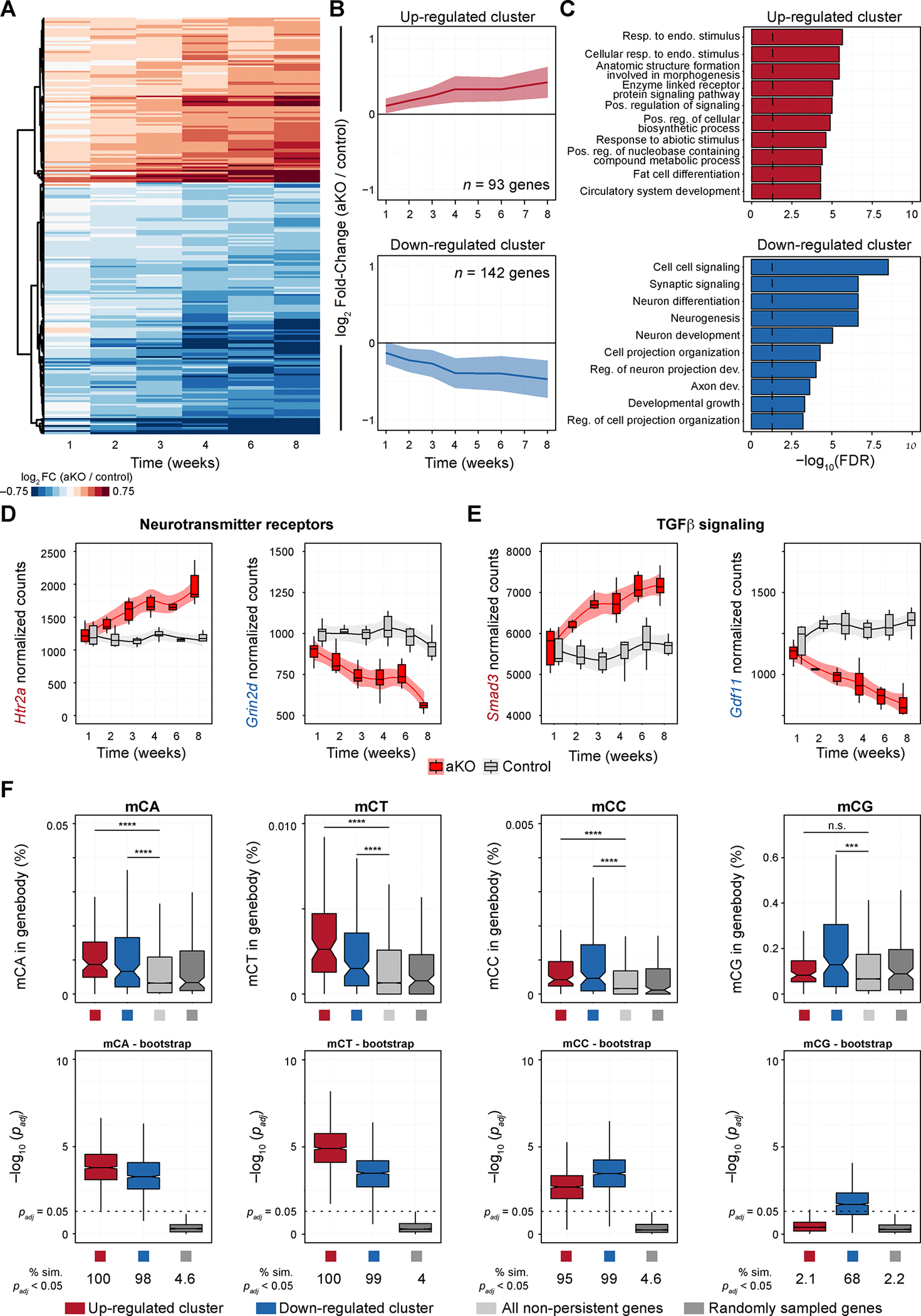
MeCP2 acutely regulates a subset of persistently dysregulated genes. (A) Heatmap of the genes persistently dysregulated after loss of MeCP2 (see Methods). (B) Summary of the up- and down-regulated cluster log_2_ Fold-Change over time. The line displays the mean ± standard deviation. (C) Top ten enriched gene ontology terms per cluster. (D) Acute up-regulation (5-hydroxytrptamine receptor 2A (*Htr2a*)) and down-regulation (glutamate ionotropic receptor NMDA type subunit 2D (*Grin2d*)) of neurotransmitter receptors upon loss of MeCP2. (E) Acute up-regulation (SMAD family member 3 (*Smad3*)) and down-regulation (Growth differentiation factor 11 (*Gdf11*)) of TGFβ signaling genes upon loss of MeCP2.(F) Persistently dysregulated genes have higher length-normalized mCH content in the gene body, as assessed using previously published methylation sequencing study^41^.Top row displays a boxplot of the percentage of methylation grouped by up-regulated, down-regulated, or non-persistently regulated genes (gray). Dark gray displays a random sample of non-persistently regulated genes of nearly equal sample size as of the persistently dysregulated genes. Bottom row displays the distribution of *p*_*adj*_-values of Kruskal-Walis test followed by Dunn’s multiple comparisons comparing up-regulated, down-regulated, or non-persistently dysregulated genes to 1000 bootstrapped simulated subsamples of non-persistently dysregulated genes (see Methods). The percentage of simulations with *p*_adj_ < 0.05 are shown below the boxplots.

With the convergence of the aKO transcriptome upon a *Mecp2* mutant-like molecular state, we next sought to explore the trajectory of transcriptional changes triggered by adult loss of MeCP2. We identified genes that robustly changed at multiple time points in the aKO samples compared to control by calculating an aggregate *p*-value per gene using Fisher’s method (see Methods; Table S4). We then removed genes that were also persistently altered in the treatment controls over time (Figure S6A). We further filtered genes that were altered by at least 20% in any direction in at least one time point. These criteria yielded 235 genes that were persistently dysregulated upon loss of MeCP2 (Figure 4A). Of these genes, 93 were upregulated and 142 were downregulated, and each set of genes had an aggregate monotonic increase or decrease in fold-change over time, respectively (Figure 4B). Loosening the Fisher’s *p*-value threshold increased the number of genes retained after filtering, but the proportion of up- and down-regulated genes remained similar (Figure S6B). We next performed gene ontology analysis to determine which pathways are persistently dysregulated. We found that the up-regulated cluster is mainly involved in cell signaling and metabolism, while the down-regulated cluster is enriched in multiple gene expression programs related to neural development and synaptic signaling (Figure 4C). These data demonstrate that acute loss of MeCP2 causes the persistent dysregulation of two transcriptional programs – one up- and one down-regulated.

Further investigation into the gene set revealed that genes within the same family could be dysregulated in opposite directions. For example, we observed the dysregulation of multiple genes in the neurotransmitter receptor family, with 5-hydroxytryptamine receptor 2A (*Htr2a*) acutely up-regulated and glutamate ionotropic receptor NMDA receptor subunit 2D (*Grin2d*) acutely down-regulated (Figure 4D). Furthermore, we identified multiple genes related to TGFβ signaling, a pathway shown to modulate MeCP2-driven pathogenesis^39^, with SMAD family member 3 (*Smad3*) acutely up-regulated, and Growth differentiation factor 11 (*Gdf11*) acutely down-regulated (Figure 4E). These data demonstrate how the loss of MeCP2 affects convergent cellular processes and pathways by both up- and down-regulating components acutely and persistently. Taken together, the identified clusters of persistently dysregulated genes are involved in neuronal function and could play a central role in pathogenesis, rather than their dysregulation being secondary to general neuronal dysfunction.

MeCP2 binds methylated cytosine within di- and tri-nucleotides, and higher non-CG methylation (mCH; H = A, C, or T) on the gene body has been shown to be a predictor of a MeCP2-regulated gene^4,21,22,40^. To determine if the persistently dysregulated genes have a distinguishable methylation context, we mined previously generated bisulfite sequencing from the hippocampus of wild-type mice of ^41^. We quantified the percentage of methylated cytosine dinucleotide context in the gene body of every gene detected in our RNA-sequencing data. We found that the persistently dysregulated gene clusters (both up- and down-regulated) have increased percentage of mCA, mCT, and mCC contexts in the gene body compared to non-persistently dysregulated genes. The down-regulated cluster had a modest increase in mCG context, which was not observed in the up-regulated cluster (Figure 4F, top row). These differences were not due to differences in the numbers of genes in each category, as we compared methylation percentages of a similar number of non-persistently dysregulated genes using bootstrap sampling (see Methods). We found that both the up- and down-regulated clusters had higher methylation content in the gene body in most subsampled simulations (Figure 4F, bottom row), demonstrating robustness in the difference in methylation content. We did not observe a similarly robust difference in methylation percentage in the promoter region, though the up-regulated cluster did have a subset of simulations that yield a significant increase in methylation in the mCC, mCT, and mCG contexts (Figure S6C, bottom row). Taken together, these data suggest that gene body methylation of mCH dinucleotides demarcate genes, both up- and down-regulated, that are robustly reliant on MeCP2 function and levels for their proper expression.

### MeCP2 is dynamically depleted from the genomic regions spanning strongly dysregulated genes

**Figure 5.**
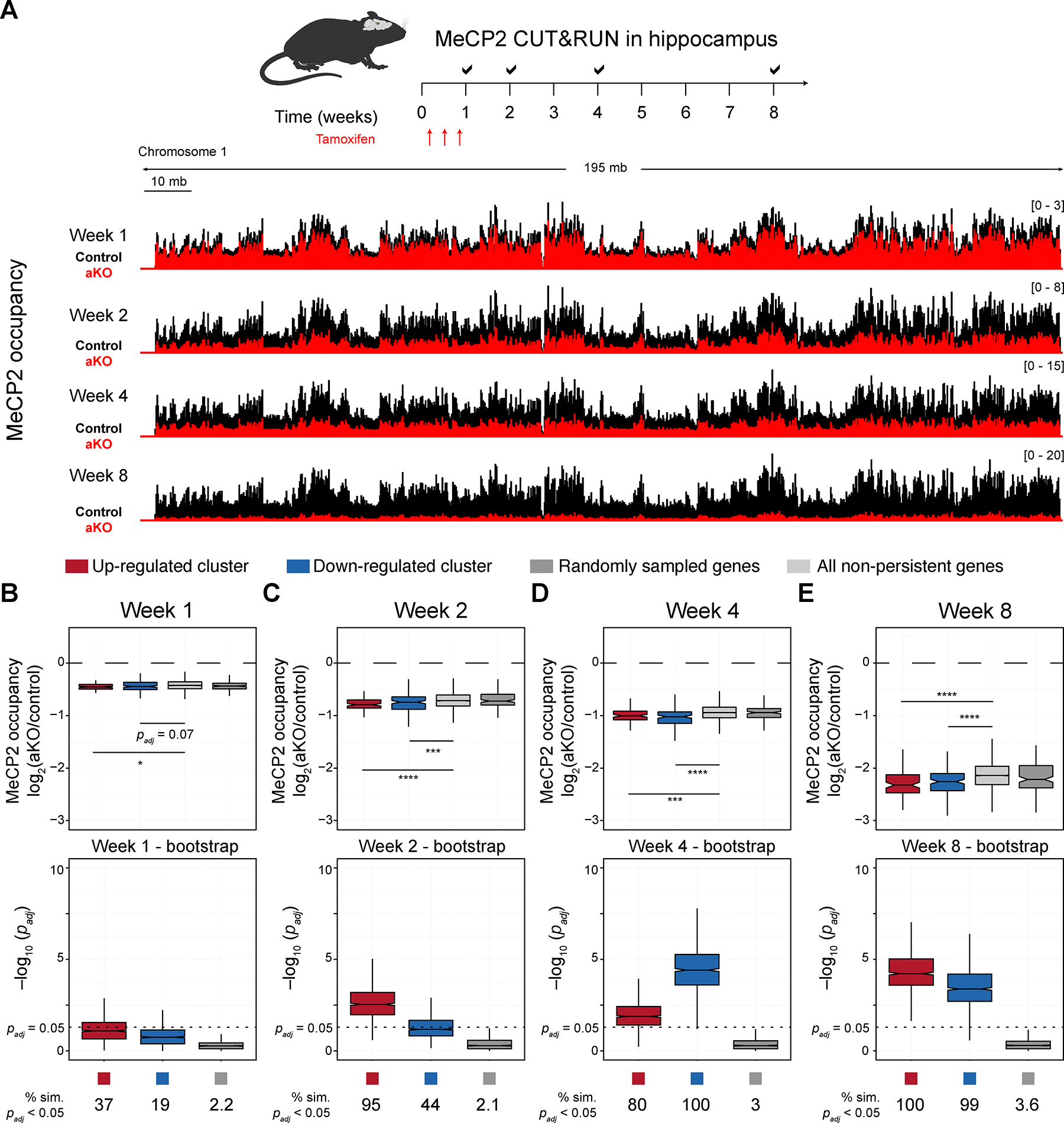
MeCP2 is depleted from persistently dysregulated genes. (A) MeCP2 binding profiles were generated at one-, two-, four-, and eight- weeks after tamoxifen treatment and deletion of *Mecp2* using CUT&RUN on hippocampal tissue collected from aKO (red) and control (black) animals. The pattern of MeCP2 binding is shown for the entire mouse chromosome 1 for each individual time point. The scale of signal intensity is shown on the upper left of each track of three pooled biological replicates. (B-E) The log_2_ ratio of MeCP2 integrated signal was tabulated for individual genes; genes were then grouped as either persistently up- or down-regulated or the background. Differences in the medians of each group was assessed by Kruskal-Wallis test followed by Dunn’s multiple comparisons correction; (***) *P* < 0.001 and (****) *P* < 0.0001. Dark gray displays a random sample of non-persistently regulated genes of nearly equal sample size as of the persistently dysregulated genes. Bottom row displays the distribution of *p*_*adj*_-values of Kruskal-Walis test followed by Dunn’s multiple comparisons comparing up-regulated, down-regulated, or non-persistently dysregulated genes to 1000 bootstrapped simulated subsamples of non-persistently dysregulated genes. The percentage of simulations with *p*_adj_ < 0.05 are shown below the boxplots.

MeCP2 binds globally throughout the genome^3,9,20,22^, but its loss has an acute consequence for just a subset of genes. We hypothesized that the depletion of MeCP2 from this subset of genes (both up- and down-regulated) may be faster leading to their acute and persistently dysregulation. To measure MeCP2 binding, we used Cleaved Under Targets and Release Using Nuclease (CUT&RUN)^39,42^. We collected nuclei from two early (one- and two-week) and two late (four- and eight-week) time points from aKO and control hippocampi and globally measured MeCP2 binding using next generation sequencing in batches by time point (Figure 5A). Overall, we observed a consistent global pattern of binding across time points in control samples, though we did observe a batch effect of absolute signal magnitude per time point. Thus, to compare the binding patterns across time points and batches, we calculated the log_2_ fold-change of MeCP2 binding between aKO and control profiles within a given time point. We first found that the average log_2_ fold-change between aKO and control profiles genome-wide was similar to what we observed by western blot (Figure 5B and Figure 1C; Table S5). These data demonstrate that CUT&RUN is quantitative and can be used to assess potentially subtle changes.

We next explored the binding of MeCP2 in the local context of expressed genes by considering the MeCP2 occupancy within a metagene that encompasses the genomic region between 2 kilobases upstream of the transcriptional start site to the transcriptional end site (see Methods). We then assessed differences in aKO versus control in genes detected by RNA-sequencing. Interestingly, we found that both the up- and down-regulated clusters of genes had greater loss of MeCP2 binding at one-week in aKO versus control (Figure 5B). This relative decrease in MeCP2 occupancy in persistently dysregulated genes was consistent across the remaining three time points (Figure 5C–E and Figure S7A). In week one, the difference in MeCP2 binding at persistently dysregulated genes was trending towards differences but not significantly different than most background gene subsamples. At week two, the up-regulated cluster had a robust decrease in MeCP2 occupancy that continued at the four- and eight-week time points. For the down-regulated cluster, a robust difference was observed beginning at four weeks after the deletion of *Mecp2*. To consolidate differences in time points, we integrated the MeCP2 binding profiles over time by fitting an exponential decay for MeCP2 loss for each gene (Figure S7A), and consistent with the static comparisons, the up- and down-regulated clusters of genes had an increased decay constant compared to all non-persistently dysregulated genes (Figure S7B). Taken together, our data suggest that MeCP2 may be depleted at a faster rate upon *Mecp2* knockout from genes that it persistently regulates.

### Loss of MeCP2 acutely alters the chromatin landscape at dysregulated genes and repetitive elements

The increased rate of MeCP2 depletion fSat persistently up-regulated genes is consistent with the hypothesis that MeCP2 binding is directly involved in repressing these genes^9,15,22,40^. However, this same notion does not explain how reduced MeCP2 binding would result in the acute down-regulation of a gene. We next sought to understand how acute loss of MeCP2 could drive gene down-regulation.

MeCP2 levels have been shown to influence the chromatin landscape, both macroscopically throughout the nucleus and locally at the level of individual loci ^9,10,12,13^. MeCP2 levels and function have been reported to be critically important to post-translational histone modifications, like acetylation, on histone H3^3,32^. To assess if the histone landscape is acutely altered upon MeCP2 depletion, we performed CUT&RUN for histone H3 acetylation (ac) and tri-methylation (me3) at lysine residues 9 and 27 in the same control and aKO nuclear preparations used to profile MeCP2 binding. These acetyl- and tri-methyl- post-translational modifications on histone H3 generally correlate with gene activation and repression, respectively^43^. The binding profiles for each histone post-translational modification were globally similar between control and aKO samples consistent with previous studies that observed only subtle differences between wild-type and germline *Mecp2*-null histone post-translational profiles^9,10^.

**Figure 6.**
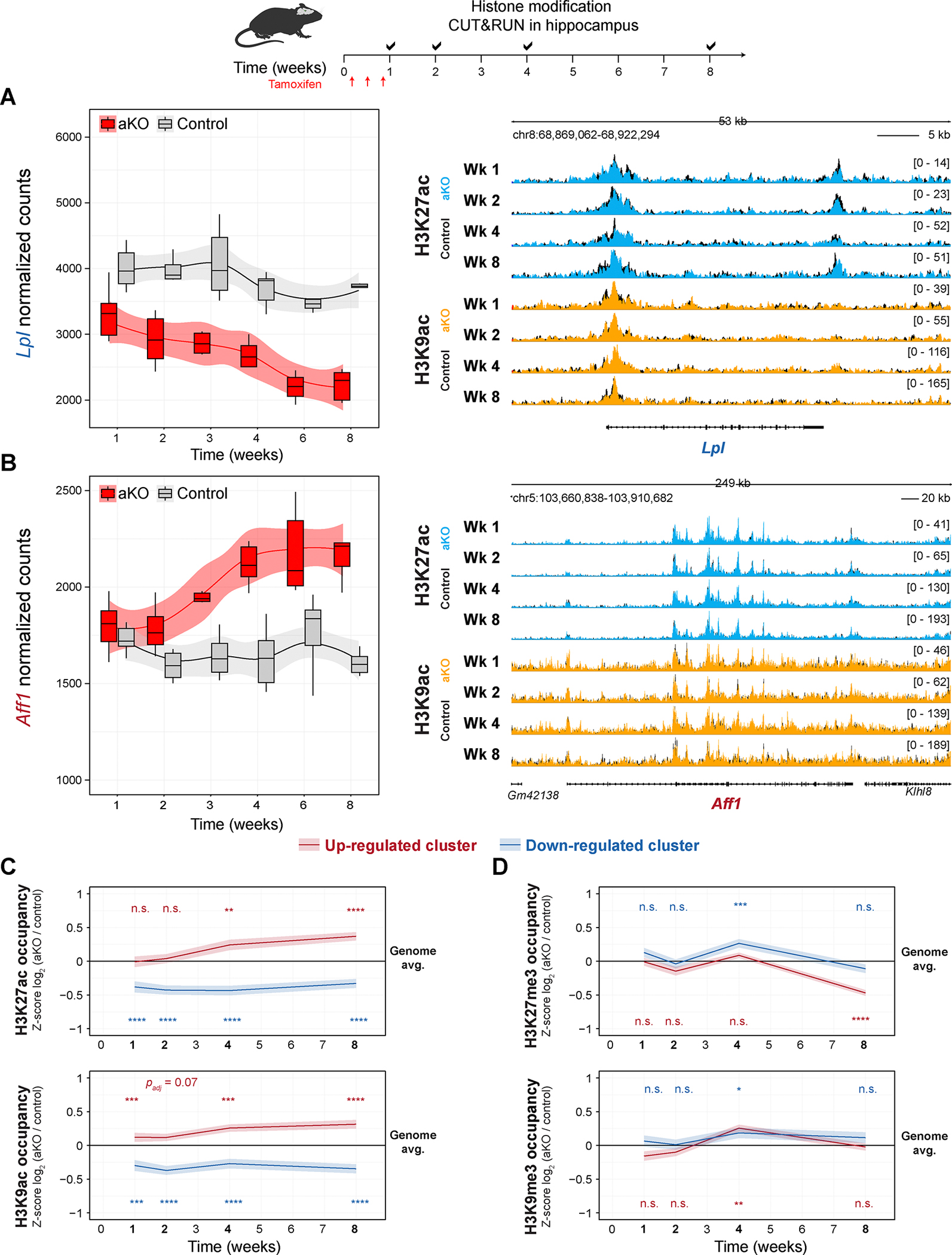
Loss of MeCP2 acutely and subtly modulates the relative chromatin landscape around persistently dysregulated genes. (A, B) Histone modification binding profiles were generated at one-, two-, four-, and eight-weeks after tamoxifen treatment and deletion of *Mecp2* using CUT&RUN on hippocampal tissue collected from aKO and control animals. The pattern of histone H3K27 and H3K9 acetylation modification is shown for the either a persistently down-regulated gene (A, *Lpl*) or up-regulated gene (B, *Aff1*). The RNA expression is displayed in the left graph, line and ribbon represent a LOESS fit to the expression values. On the right, tracks from aKO tissues (cyan and orange for H3K27ac and H3K9ac, respectively) are overlayed over the matched control samples (black). The scale of signal intensity is shown on the upper left of each track from three pooled biological replicates. (C, D) The Z-score log_2_ fold-change of integrated density of a histone mark between aKO and control samples was tabulated for each gene and grouped by either up-regulated, down-regulated, or non-persistently dysregulated genes upon loss of MeCP2. *p*-values were determined using Kruskal-Walis test followed by Dunn’s multiple comparisons comparing up-regulated, down-regulated, or non-persistently dysregulated genes (see Figure S9); (***) *P* < 0.001 and (****) *P* < 0.0001. (C) Pattern of H3K27ac and H3K9ac in each group. (D) Pattern of H3K27me3 and H3K9me3 in each group.

Next, we sought to quantitatively compare binding signals locally. We first observed a subtle trend towards a decreased histone H3 acetylation profile for down-regulated genes and a subtle trend towards an increased histone H3 acetylation profile for up-regulated genes compared to the background genome (Figure 6A, B and Figure S8). To quantify these subtle differences, we integrated the signal of each histone modification from promoter (−2 kb) to transcriptional end site for each gene detected by RNA-sequencing and calculated the log_2_ fold-change between genotypes^9,10^ (see Methods; Figure 9A-D and Table S6). With three biological replicates per genotype per time point, we did not have the power to identify significant differences (*p*_adj_ < 0.05) at individual gene bins, further emphasizing the subtle changes. We then asked if the clusters of persistently up- and down-regulated genes had a shift in histone post-translational occupancy by comparing the distribution of fold-changes amongst the two clusters compared to the background non-persistently dysregulated genes. For up-regulated genes, we saw a gradual accumulation of acetylation that was pronounced at both lysine residues by four weeks (Figure S9A,B) and observed a decreased methylation at lysine residue 27, but not lysine residue 9, at later time points, generally aligning with a chromatin landscape leading to up-regulation of the gene (Figure 9C,D). In contrast, the down-regulated genes had a diminished acetylation at both histone H3 lysine residues from the earliest time point assessed, and this trajectory was not matched with a concordant increase in histone methylation (Figure S9A–D). These relative changes were also not present in analysis of non-targeting IgG signal, except for a modest increase in up-regulated genes at the final eight-week time point (Figure S9E). We attribute this modest increase to a reflection of overall chromatin accessibility for these strongly up-regulated genes^42^. We performed bootstrapped replicates comparing the distribution of fold-changes of the up- and down-regulated clusters to random subsamples of a subset of non-persistently dysregulated genes and found that the observed differences were robust to gene set sample size (Figure S9). Taken together, our data demonstrate that loss of MeCP2 in adulthood results in a subtle change of the local chromatin landscape, particularly neighboring genes persistently dysregulated upon loss of MeCP2.

To collate the series of experiments together and overcome batch effects, we centered the normalized log_2_ fold-change signal intensity within each time point to generate a trajectory of histone signal for each category of genes. For up-regulated genes, we observed a small increase in H3K27ac at early time points and a small, but significant, increase in H3K9ac at early time points that progressively increased over time after loss of MeCP2 (Figure 6C). We observed a significant decrease in H3K27me3 only at the end time point and no consistent trajectory for H3K9me3 (Figure 6D). In contrast, for down-regulated genes, we observed a significant relative decrease in histone acetylation even at the earliest time point (Figure 6A,C). These data suggest that the local chromatin state reflects the changes in transcription acutely upon loss of MeCP2.

Given that loss of MeCP2 has been linked to increased transcription from LINE-1 elements^7,8,44^, we measured the occupancy of MeCP2 and the histone modifications specifically at repeat elements throughout the genome grouped by repeat type (see Methods). Surprisingly, we found that MeCP2 occupancy at repeat elements was significantly, though subtly, elevated in the aKO model at one-week. MeCP2 then is lost with similar kinetics as the regions surrounding all coding genes at the remaining time points, culminating with a ~90% reduction in MeCP2 occupancy at the eight-week time point. These data suggest that the trajectory of MeCP2 depletion differs at repeat regions compared to genes in the early, but not later, time points (Figure S10A). We then evaluated the histone landscape at these same regions and found that histone H3K9me3 also exhibited a significant increase at the one-week time point, and subsequent decrease at weeks two and four, culminating at a decrease at week eight (Figure S10B). Importantly, H3K9me3 occupancy at repeat regions is critical for the maintenance of heterochromatin^45,46^. This decrease in H3K9me3 coincided with a subtle increase in H3K9ac ( Figure S10C). We observed a qualitatively similar early increase and later decrease in histone H4K20 di/tri-methylation, though the pattern was not monotonically consistent^47^ and no trajectory in histone H3K27ac ( Figure S10D,E and Table S7). Our data indicate that acute loss of MeCP2 leads to its redistribution or possible retention at repetitive elements at early time points prior to its complete depletion. This transient change in MeCP2 to repetitive elements is accompanied by an increase in the repressive histone mark (H3K9me3), but the chromatin state opens around repeat regions over time after MeCP2 depletion. These patterns were preserved when considering whether the repeats were located within the heterochromatin or euchromatin (Figure S10A–E), though more robustly captured in the heterochromatin due to the overall increased representation of repeat reads from heterochromatic regions in our epigenomic profiles (Figure S10F). More broadly, taken together, our data demonstrate that MeCP2 levels help to maintain the normal histone landscape in the adult brain that rapidly, but subtly, goes awry upon MeCP2 depletion.

### Electrophysiologic and behavioral deficits progressively arise upon loss of MeCP2

**Figure 7.**
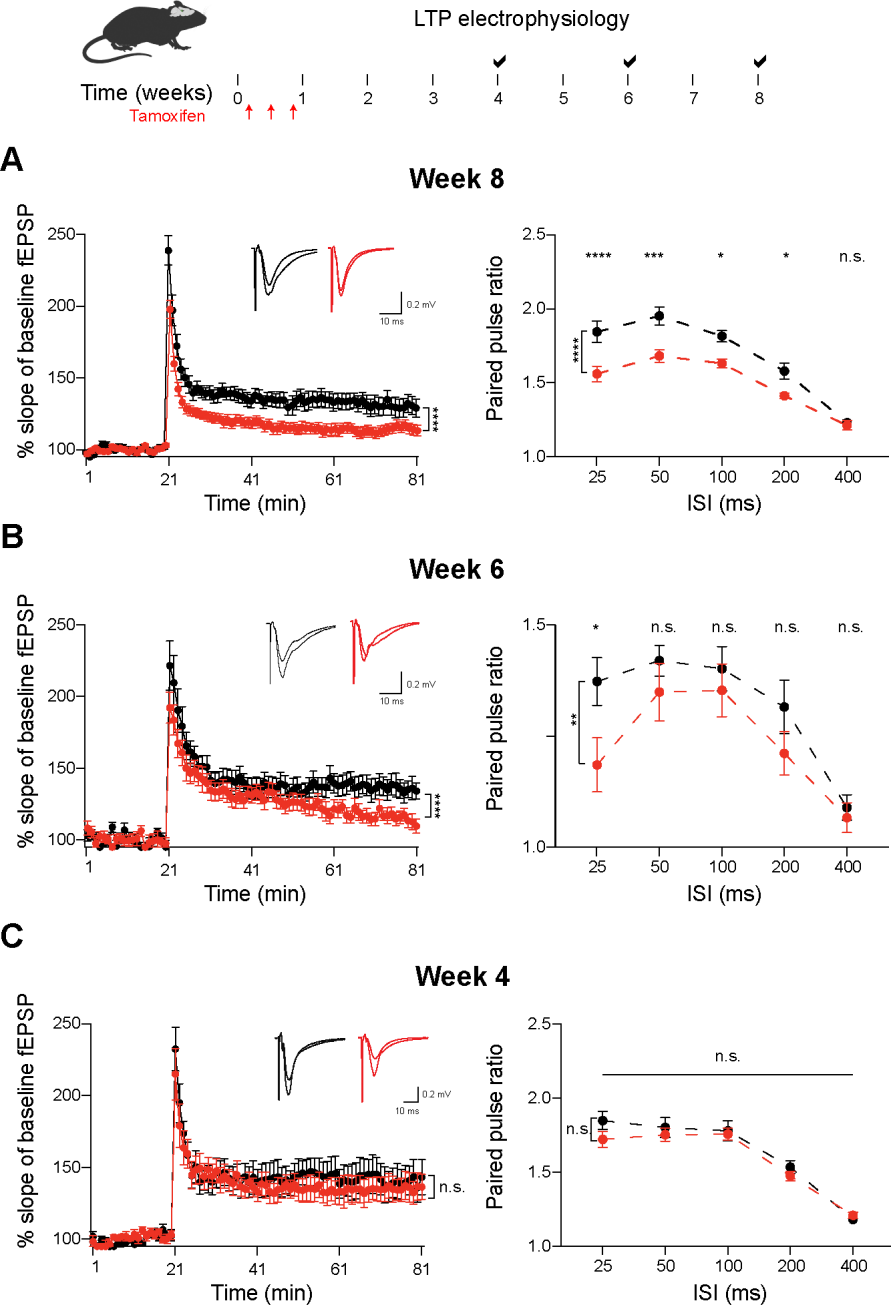
Hippocampal electrophysiologic deficits lag behind depletion of MeCP2 protein. Mice were treated with vehicle or tamoxifen (TMX) as described in Figure 1. (A-C) Left, Long-term potentiation (LTP) at eight- (A), six- (B), or four- (C) weeks after *Mecp2* deletion. Inset graphs show representative electrophysiological traces at baseline and high-frequency stimulation. Right, pair-pulse ratio at eight- (A), six- (B), and four- (C) weeks after *Mecp2* deletion (*n* = 6–7 animals per condition per time point, *N* = 12–30 slices per condition per time point). Data were analyzed with two-way ANOVA and *p*-value denotes genotype comparison (****) *P* < 0.0001; specific bins of paired pulse ratio were assessed after multiple comparisons correction; (*) *P* < 0.05, (***) *P* < 0.001, (****) *P* < 0.0001.

Deletion of *Mecp2* in adult mice leads to behavioral decline reminiscent of *Mecp2* germline-null mice^28–31^. Furthermore, germline loss of MeCP2 causes electrophysiologic and circuit-level deficits^24^. We sought to determine if electrophysiologic deficits preceded the global molecular dysregulation in the hippocampus to exclude a decline in neuronal activity, and not MeCP2, as driving the transcriptional and epigenetic changes. We measured long-term potentiation (LTP) in the hippocampus of aKO and control mice starting at the terminal 8-week time point where we would expect a phenotypic deficit. Indeed, at 8-weeks post *Mecp2* deletion, we observed a robust deficit in LTP and a decrease in the measured interspike interval (Figure 7A). We then performed LTP at earlier time points until no deficits were observed. At 6-weeks we observed a significant (*p* < 0.01) decrease in LTP and a mild decrease in measured interspike interval (Figure 7B). Neither of these deficits was observed in measurements performed in 4-week post *Mecp2* deletion hippocampi (Figure 7C). Importantly, the number of DEGs is similar in both the 4- and 6-week time points (Figure 2B), suggesting that circuit-level deficits occur after global transcriptional dysregulation upon loss of MeCP2.

We next assessed the timing of the behavioral deficits induced by loss of MeCP2. We first used weekly weights as a general assessment of animal health. We observed that CreER activitation caused weight gain in the aKO and Cre-only treatment controls for the first two weeks (Figure S11A). However, after three weeks, only the aKO mice showed significant (*p* < 0.0001) weight gain compared to control and treatment control mice (Figure S11A). These data demonstrate that weight is a sensitive measurement reflective of acute changes to MeCP2 protein level controlled by the hypothalamic circuits in mice^48^.

Beyond organism physiology, we then used quantitative behavioral assays to assess the time point at which neurological deficits are observed. Importantly, we assessed behavior at each time point using an independent cohort of mice as repeated behavioral training can overcome behavioral deficits induced by MeCP2-deficiency^49^. At eight-weeks after *Mecp2* deletion, we observed aKO mice were significantly hypoactive in the open field assay with decreased distance traveled (*p* < 0.0001) and horizontal activity counts (*p* < 0.001). The decrease in distance traveled was detected at four weeks, while the decrease in exploratory activities was detected at six weeks (Figure S11B,C). Next, we assessed hippocampal function by performing fear conditioning using a tone-shock paradigm. However, due to the severe hypoactivity and weight gain, we were unable to rigorously assess learning and memory using this assay as hypoactivity was scored as freezing and learning. At six- and eight-weeks post-deletion, aKO mice were frozen for a significantly (*p* < 0.0001) longer time than control mice at baseline (Figure S11D, E). We did not observe any behavioral deficits in either the open field or conditioned fear assays in treatment controls (Figure S11B–E), which demonstrates that the phenotypic deficits observed are specific to the loss of MeCP2. Taken together, our data demonstrate that transcriptional and epigenomic changes resulting from the loss of MeCP2 occur before behavioral and physiologic deficits manifest.

## DISCUSSION

Loss-of-function mutations in *MECP2* cause Rett syndrome, and previous work has uncovered that loss of MeCP2 protein function causes a myriad of molecular, cellular, and physiologic abnormalities in the brain. Despite the devastating physiologic consequences of reduced MeCP2 protein function, the molecular changes are surprisingly mild in magnitude^50^. Furthermore, the broad, yet subtle, molecular changes that occur upon loss of MeCP2 function have been difficult to deconvolute from developmental changes or secondary consequences consequential to neuronal dysfunction. In this study, harnessing that MeCP2 function is required throughout life^28–31^, we deleted *Mecp2* in adult mice at a time point where MeCP2 function is critically important^29–31^. We performed a time series of RNA-sequencing, chromatin, electrophysiological, and behavioral measurements to track the cascade of molecular and phenotypic events that occur as MeCP2 is lost.

### Insights into molecular mechanisms of MeCP2-driven pathogenesis

By removing the considerations of development and secondary pathogenesis, we revealed a core set of transcripts acutely and persistently regulated by MeCP2. Many of these genes are directly related to neuronal function, and some of these genes have been directly shown to modulate MeCP2-driven pathogenesis, like *Gdf11*^39^. We found that both the persistently up- and down-regulated genes were highly methylated at mCH sites in the gene body, corroborating other studies that have demonstrated that genes altered in *Mecp2* germline knockout transcriptomes are highly methylated^9,10,20–22,40^. Interestingly, this increased methylation content corresponded to a faster loss of MeCP2 binding locally within the persistently dysregulated genes, as assessed by the depletion rate of MeCP2 binding *in situ*.

Recent studies using single particle imaging revealed that MeCP2 does not statically bind the genome. MeCP2 is constantly binding and releasing methylated DNA globally throughout the nucleus^51^. Our CUT&RUN data suggest that this binding may have different kinetics locally within the genome, with MeCP2 moving dynamically at genes it most robustly regulates. For up-regulated genes, this may involve constant cycling of MeCP2 at the gene body to repress the expression of these genes, potentially by inhibiting RNA polymerase or RNA polymerase initiation or processivity^9,52^. Beyond this initial direct repression, loss of MeCP2 may prevent the recruitment of HDACs that would otherwise normally maintain lower expression for these up-regulated genes. We observed an increase in H3K9 and H3K27 acetylation for the up-regulated genes at 4-weeks post *Mecp2* deletion, consistent with a role for MeCP2 recruiting HDACs at these loci in the normal brain^15^. For down-regulated genes, we observed that loss of MeCP2 function causes the immediate relative decrease in histone acetylation in the local context of these down-regulated genes. We speculate that this constant movement and recruitment of MeCP2 may critically maintain the local chromatin landscape, particularly the balance of histone acetylation at a given locus, to keep these genes activated in the brain^53^. MeCP2 has been suggested to also repress expression at repeat elements. Surprisingly, we observed a transient increase of MeCP2 and H3K9me3 occupancy at these regions early after *Mecp2* deletion, suggesting that MeCP2 may be preferentially retained at repeats compared with its rapid turnover at genes. How the chromatin landscape at repeat regions influences gene expression is poorly understood^54^. Further work is needed to understand if MeCP2 has preferential binding to local repeat elements and to understand whether these differential retention dynamics have any influence on transcriptional regulation of nearby genes^54^. Towards this goal, additional future work combining imaging with markers for the MeCP2-responsive genomic regions will be able to directly associate the transcriptional changes identified in this study to single molecule dynamics of MeCP2.

A key finding from this study is that electrophysiologic, circuit-level deficits in the hippocampus occurred after transcriptional dysregulation. The data suggest that transcriptional dysregulation occurs upon *Mecp2* deletion, and these changes contribute to reduced neuronal function. Our data also provide a resource to identify genes dysregulated downstream of MeCP2, but upstream of electrophysiologic deficits, that are critical for proper neuronal function. We identified a set of neurotransmitter receptors, *Grin2d* and *Htr2a* that are acutely dysregulated upon loss of MeCP2. These genes warrant further study to determine their dosage-sensitivity to neuronal function. Lastly, our data demonstrate that there is a window of time where molecular events downstream of MeCP2 are occurring, but before overt phenotypic consequences are measurable. Investigating the cell-type specific changes during this window including the cell autonomous versus non autonomous effects might explain some of the glial responses and will be important in fully characterizing the trajectory of molecular events.

### Acute perturbations of dosage-sensitive genes

MeCP2 is the exemplar dosage-sensitive gene, whose precise levels need to be maintained throughout life for proper neuronal function^28–31^. There are additional dosage-sensitive genes that are required throughout life like cyclin-dependent kinase-like 5 (*CDKL5*), the causative gene for CDKL5 deficiency disorder^55,56^. Depletion of *Cdkl5* in adult mice causes the onset of several behavioral and neurophysiologic deficits, analogous to the onset of deficits observed upon adult depletion of *Mecp2*^57^. However, the underlying molecular cascades downstream of CDKL5 have not been enumerated. Our study provides a framework and roadmap for how to utilize adult depletion of dosage-sensitive genes to identify programs of genes acutely regulated by the disease-causing gene. Our data catalog the limited non-specific tamoxifen and CreER recombinase related molecular effects that can be disregarded in future studies utilizing floxed alleles to induce adult depletion of disease-causing genes. In doing so, we can identify the gene programs most proximal to several disease-causing genes, especially gene programs dysregulated as neuronal function declines, and shed new light into pathogenesis of multiple disorders.

### Therapeutic implications

MeCP2 function is required throughout life, and thankfully, postnatal restoration of MeCP2 function can rescue neurological dysfunction in a mouse model of RTT^58^. This seminal work has laid the foundation for pursuing gene therapy strategies to treat RTT^59–61^. The challenge in using gene therapy approaches with Rett syndrome, is that MeCP2 is a dosage-sensitive gene and too much MeCP2 expression is toxic for brain function causing *MECP2* duplication syndrome (MDS)^62,63^. Conversely, for MDS, antisense oligonucleotide therapy to reduce *MECP2* expression is an attractive treatment option^34,35^. However, with ASO treatment of MDS, we must be careful to not overly reduce MeCP2 towards RTTlike levels. These considerations have highlighted the importance of having molecular markers that are reflective of MeCP2 levels. Interestingly, some of these markers are secreted proteins – like the TGFβ ligand *Gdf11*^39^. Thus, our data can be leveraged to identify genes not only involved in pathogenesis, but that could also serve as proximal biomarkers reflective of MeCP2 protein levels.

Our data also revealed that various behavioral and physiologic changes are altered at different times after loss of MeCP2. For example, weight is altered within three weeks after loss of MeCP2, while locomotive deficits arise after four weeks. Within the hippocampus, circuit-level electrophysiologic deficits manifest even later. These data suggest that brain region vulnerability to loss of MeCP2 function may vary. The increase in weight gain, which mirrors the hypothalamus specific knockout of *Mecp2*^48^, could indicate that the hypothalamus either has an increased reliance on MeCP2 for normal function or perhaps MeCP2 is depleted from this region at a faster rate than in other regions of the brain. Future studies dissecting the chronology of multiple behavioral areas will be important to understand which phenotypes could be altered earlier than others as MeCP2 protein levels are depleted. These could be phenotypes that are more sensitive to MeCP2 expression in the clinic and an important outcome measurement of a tested treatment. In this case, potentially weight, coupled with a molecular marker, could inform if ideal MeCP2 levels have been therapeutically achieved, highlighting the importance of cataloging the cascade of events that occur downstream of changes in MeCP2 levels and function.

## RESOURCE AVAILABILITY

### Lead contact

Further information and requests for resources and reagents should be directed to and will be fulfilled by the lead contact, Dr. Huda Y. Zoghbi (hzoghbi@bcm.edu).

### Materials availability

Except for the *Mecp2*-G118E mouse line, all other materials are available commercially. *Mecp2*-G118E mice can be requested from the Lead Contact.

### Data and code availability

All sequencing data generated in this study or re-analyzed in this study is available on GEO. Western blot data is deposited to Zenodo. This paper reports no original code; any materials needed to analyze data from this manuscript can be requested from the Lead Contact.

## STAR METHODS

### Animals

#### Breeding and colony maintenance

Baylor College of Medicine Institutional Animal Care and Use Committee (IACUC, Protocol AN-1013) approved all mouse care and manipulation. Mice were housed in an AAALAS-certified level 3 facility on a 14-hour light cycle. Mice were monitored daily by veterinary staff. All procedures to maintain and use these mice were approved by the Institutional Animal Care and Use Committee for Baylor College of Medicine and Affiliates. To generate *Mecp2* adult knockout males, *Mecp2*^*flox*/+^ (*Mecp2*^*tm1Jae*^; RRID:MMRRC_011918-UCD) female mice were bred with *UBC-CreERT2* (RRID: IMSR_JAX:007001) male mice. *Mecp2*^*G118E/y*^ point mutation mice were generated by breeding *Mecp2*^*G118E*/+^ females to C57BL6/J wild-type mice (RRID: IMSR_JAX:000664) as previously described ^38^. All mice were maintained on a C57BL6/J background. Investigators were blind to genotype until after either tissue were collected or behavioral analyses performed.

#### Tamoxifen treatment

Tamoxifen (Sigma-Aldrich #T5648) was resuspended in peanut oil vehicle (Sigma-Aldrich #P2144) at a concentration of 20 mg/mL and stored at −20°C until use. Flox; Cre, Flox only, Cre only, and wild-type littermates were treated with tamoxifen at a dose of 100mg/kg delivered intraperitoneally every other day for one week for a total of three treatments. A subset of Flox; Cre littermates were treated with peanut oil delivered intraperitoneally every other day for one week for a total of three treatments. Tamoxifen-treated animals were not housed together with vehicle peanut oil treated animals.

### RNA extraction, reverse transcription, and qPCR

Total RNA was isolated from one hippocampus using the Qiagen miRNeasy Mini kit (Qiagen #217004). For *Mecp2*^*G118E/y*^ samples, total RNA was isolated from one frontal cortex using the Qiagen miRNeasy Minit kit as previously described^36^. On column DNAse digestion (Qiagen #79254) was performed according to manufacturer’s protocol to remove genomic DNA. 2 μg of total RNA was used to synthesized cDNA using the M-MLV reverse transcriptase (Life Technologies) according to manufacturer’s protocol. qRT-PCR was performed using a CFX96 Real-Time System (Bio-Rad) using PowerUp SYBR Green Master Mix (ThermoFisher #A25741), 0.4 μM forward and reverse primers, and 1:20 dilution of cDNA. The following cycling conditions were used: 95°C for 5 min, 39 cycles of 95°C for 11 sec, 60°C for 45 sec, plate read, a final melt of 95°C, and melt curve of 65–95°C at +0.5°C increments. The specificity of the amplification products was verified using melt-curve analysis. The Ct values were calculated with the Bio-Rad software, and relative gene expression was calculated using the ΔΔCt method using *Ppia* for normalization. All reactions were performed in technical duplicate with a minimum of three biological replicates. Data are presented as mean ± SEM in main figure.

#### qPCR primer sequences

**Table:** 

Primer name	Sequence (5’ – 3’)
*Ppia* forward primer	GCATACAGGTCCTGGCATCT
*Ppia* reverse primer	CCATCCAGCCATTCAGTCTT
*Mecp2* forward primer	TATTTGATCAATCCCCAGGG
*Mecp2* reverse primer	CTCCCTCTCCCAGTTACCGT

### RNA-sequencing and analysis

#### Library preparation, next generation sequencing, RNA-sequencing alignment

RNA was isolated as described above and sent to Genewiz for RNA integrity assessment, library preparation, and sequencing on the Illumina HiSeq platform. For each sample, approximately 100 million 150bp pair-end reads were generated. Raw reads were trimmed before mapping by Trimmomatic v0.39 using the default parameters^64^. Trimmed reads were aligned to GRCm38.p6 assembly from GENCODE with the addition of the human *MECP2* sequence using STAR v2.7.9.d using all default parameters except –sjdbOverhang149^35,65^. One tamoxifen-treated, Cre only sample from the first week time point was excluded from further analysis due to outlier gene body coverage and increased presence of overrepresented sequences.

#### Differential gene expression analysis of *Mecp2* adult-knockout transcriptomes

The read counts were analyzed for differential gene expression using the DESeq2 package v.1.34.0^66^. Only genes with an average count above 10 across all samples were retained for further downstream analyses. All read counts were normalized together within the same DESeq structure, but specific differentially expressed genes were extracted in as pairwise comparisons between conditions at one time point (e.g., Flox;Cre+TMX vs. Flox;Cre+Vehicle at 1-week or WT+TMX vs. Flox;Cre+Vehicle at 1-week). Genes were counted as differentially expressed if the adjusted *p*-value was below 0.05, the absolute value of log_2_ fold-change versus Flox;Cre+Vehicle was above 0.15 (corresponding to ~10% change), and a standard error below 0.5. For each time point, the differentially expressed genes between Flox;Cre+Vehicle and the treatment controls (WT, Flox, Cre+TMX) were tabulated. These genes were subtracted from the final gene set of differentially expressed genes between Flox;Cre+TMX and Flox;Cre+Vehicle for any given time point. Principal component analysis was performed using the DESeq2 PCA function. Overlap between differentially expressed genes at each time point was assessed using the GeneOverlap package in Bioconductor.

Genes consistently altered over time upon loss of MeCP2 were identified by aggregating the *p*-adjusted value from each individual time point analysis by calculating Fisher’s combined *p*-value using the poolr v1.1–1 package in R. Genes were retained as persistently dysregulated in the treatment controls if their Fisher’s combined *p*-value was less than 0.1 and that gene was altered by at least 20% in at least one time point. Genes were retained as persistently dysregulated in the adult knockout time series if their Fisher’s combined *p*-value was less than 1×10^−5^ and that gene was altered by at least 20% in at least one time point. Genes that were persistently dysregulated in the treatment controls (Fisher’s *p*-value < 0.1) were removed from the set of genes persistently dysregulated in the adult knockout.

#### Comparison of *Mecp2* adult-knockout transcriptome to germline *Mecp2*-null transcriptome

Gene expression measurements of germline *Mecp2*-null male mice as assessed by RNA-sequencing were mined from five previously published studies and from *Mecp2*^*G118E/y*^ RNA-sequencing generated in this study^9,10,20,36,37^. The raw sequencing data was obtained from the Gene Expression Omnibus or obtained from Genewiz as described above and re-aligned/aligned to the mouse genome in a previously published meta-analysis^50^. The read counts were then analyzed for differential gene expression between the wild-type and germline *Mecp2*-null samples in each study using the DESeq2 package v.1.34.0^66^. Each study was processed and analyzed separately. Differentially expressed genes were called using an adjusted *p*-value threshold of 0.05, an absolute log_2_ fold-change between *Mecp2*-null and wild-type of 0.15, and a standard error less than 0.5. Overlap between differentially expressed genes at each time point was assessed using the GeneOverlap v.1.28.0 package in Bioconductor.

#### Cell-type deconvolution analysis

To deconvolute the bulk RNA-sequencing results by cell type, we utilized two publicly available single-nucleus RNA-sequencing (snRNA-seq) datasets: GSE143758^67^ and GSE234278^68^. From GSE143758, three wild-type samples from the 7-month age time point were selected, while from GSE234278, four wild-type samples from the 3-month age time point were selected. The raw counts from these datasets were processed using the SCTransform function from Seurat v.4.3.0^69^, followed by integration with the Harmony algorithm v.0.1.1 to account for batch effects^70^. Specific markers were used to determine the cell types within the integrated snRNA-seq clusters: 1) excitarory neurons – *Slc17a7* and *Camk2a*, 2) inhibitory neurons – *Gad1* and *Gad2*, 3) oligodendrocyte – *Mog* and *Mbp*, 4) oligodendrocyte precursors (OPC) – *Pgdfra*, *Cspg4*, and *Sox10*, 5) astrocyte – *Slc1a3*, *Aqp4, Fgfr3*, *Glul*, *Slc1a2*, and *Gfap*, 6) microglia – *Ptprc*, *Cx3cr1*, *Csf1r*, and *P2ry12*, 7) fibroblast – *Slc6a13*, 8) endothelial – *Flt1*, and 9) pericyte – *Vtn*^67,68^. Fibroblast, endothelial, and pericyte populations were excluded from the deconvolution analysis. The CIBERSORTx algorithm (https://cibersortx.stanford.edu) was employed to build a single-cell signature matrix based on the integrated snRNA-seq data^71^. Concurrently, the counts per million (CPM) values from the bulk RNA-seq samples were calculated using edgeR v.3.38.4^72^.

The “Input Cell Expression” module in CIBERSORTx, using the “high-resolution” option enabled, was used to estimate the cell-type proportions and calculate cell-type expression profiles from the bulk RNA-seq samples. A union of DEGs from multiple time points, defined by *p*_*adj*_ < 0.01 and log_2_ Fold-Change > 0.25, was used as the gene subset for analysis. To identify cell-type specific DEGs, a Wilcoxon test was performed by comparing Flox;Cre+TMX samples to Flox; Cre+Vehicle samples using the deconvolved expression values. A *p*-value of 0.05 with this analysis was considered statistically significant between groups.

#### Gene ontology analysis

Gene ontology analysis of the DEGs at each time point was performed using the HOMER toolbox v.4.11.1^73^. The top 10 significant (*p* < 0.05) gene ontology biological process terms were extracted per query and the −log_10_ *p*-value per term were plotted. Gene ontology analysis of the persistently dysregulated genes was performed by inputting the gene set of interest into the GSEA portal (www.gsea-msigdb.org). The top 10 significant (*p*_*adj*_ < 0.05) gene ontology biological process terms were extracted per query and the −log_10_ *p*-adjusted value per term were plotted.

### Methylation sequencing re-analysis

Raw Reduced representation bisulfite sequencing (RRBS) data was obtained from the Gene Expression Omnibus (GEO) database under accession number GSE85251^41^ using SRA (Sequence Read Archive) toolkit^74^. The dataset included multiple replicates of hippocampal tissue obtained from adult 20-week C57BL6/J wild-type mice. Raw, paired-end, reads were aligned to a bisulfite genome using Bismark v0.23.1^75^. The bisulfite genome was created using the bismark_genome_preparation command with the GENCODE GRCm38 release 23 FASTA file. Alignments from each replicate were combined into individual BAM files using the merge command from samtools v1.10^76^. Methylated cytosines were called in three different contexts (CpG, CHG, and CHH) using the bismark_methylation_extractor command, and this output was converted to bedgraph format using bismark2bedGraph script. Methylation calls were processed to generate specific bedgraph files for CAH, CTH, CGH, and CCH contexts, based on the A, T, G, or C denoted in the second position of the context sequence. These bedgraphs were sorted using bedtools v2.27.1^77^ and converted to bigwig files using the bedGraphToBigWig utility^78^. Methylation levels of genes at specific annotations were computed from the bigwig files using the pyBigWig package of deeptools and the GENCODE GRCm38 release 23 coordinates^79^. The methylation signal was quantified in the gene body between transcriptional start and end sites and in the promoter as defined as 2 kb upstream of the transcriptional start site. The signals per gene were normalized to overall gene length and compared across groups defined by gene expression changes. The differences in methylation by region were grouped by cluster identity and compared using Kruskal-Wallis test with Dunn’s post hoc test using the kwAllPairsDunnTest function in the PMCMRplus v.1.9.6 package in R. Robustness of global trends were assessed using bootstrapped subsamples of random background genes (see below).

### CUT&RUN epigenetic profiling

#### Nuclear isolation

Nuclei were isolated from frozen hippocampi from adult-knockout and control littermates (*n* = 3) using an iodixanol gradient modified from^80^. Briefly, three flash frozen hippocampi from multiple animals of the same genotype and time point were pooled and dropped into a 7 mL dounce homogenizer containing 5 mL buffer HB (0.25 M Sucrose, 25 mM KCl, 2 mM Tricine KOH pH 7.8, 500 μM Spermidine) and dounced 10x with loose pestle A and 20x with tight pestle B. Then 320 μL of HB-IGEPAL (HB Buffer + 5% IGEPAL CA-630) was added to each homogenizer and dounced 20x more with tight pestle. Each sample was incubated for 10 mins on ice and filtered through a 30 μM filter into a conical tube containing 5ml of iodixanol working solution (5 volumes Optiprep (Sigma Aldrich, D15556) + 1 volume Optiprep Diluent (150 mM KCl, 30 mM MgCl_2_, 120 mM Tricine-KOH pH 7.8)) and mixed by tube inversion.

To set up the gradient, 4 mL of 40% Iodixanol (3 volumes working solution + 1 volumes HB buffer) was added to a 50 mL round bottomed conical tube. Then, 7.5 mL 30% Iodixanol (3 volumes working solution + 2 volumes HB) was slowly overlayed on top, followed by 10 mL of the sample containing mixture prepared above. This gradient was spun at 10,000g for 20 mins at 4°C in a hanging bucket centrifuge (Sorvall Lynx 6000) with “decel” turned off. After centrifugation, nuclei are located at the interface between 30% and 40% iodixanol layers. Iodixanol containing supernatant above the nuclei was slowly discarded with bulb pipette. Approximately 4 mL of the interface containing nuclei were collected and placed into 15 mL conical tube.

The number of nuclei were quantified by taking 20 μl of sample and mixing it with 2 μL 0.2 mg/mL DAPI diluted in HB buffer. After three-minute incubation at RT, the nuclei were diluted 1:10 in buffer HB and counted on a Countess II with DAPI to quantify.

#### CUT&RUN epigenetic profiling

Cleavage Under Targets & Release Nuclease (CUT&RUN) was performed on nuclei isolated from above following^42^. Briefly, we performed one nuclear isolation per sample from a pool of three frozen hippocampi, and then split the nuclei into six individual tubes for the antibodies surveyed (MeCP2, H3K27me3, H3K27ac, H3K9me3, H3K9ac, and IgG). An individual sample will refer to one antibody from a unique pool of hippocampi.

To activate Concanavalin A coated magnetic beads (Bangs Laboratories Inc., #BP531) for binding, we incubated 25 μL of beads per sample with 3x volumes binding buffer (20 mM HEPES-NaOH 7.5, 10 mM KCl, 1 mM CaCl_2_, 1 mM MnCl_2_), rotated at RT for 5 minutes, and washed 2x with 1 mL binding buffer. All washes are done by placing microcentrifuge tube on magnetic rack and waiting until solution is clear as beads separated from the solution. Following washes, the beads were resuspended in 100 μL binding buffer per sample.

After bead activation, 900 μL beads were added to 1.8×10^6^ nuclei in the iodixanol mixture from each nuclei preparation (total volume 3.9 mL) and rotated for 10 minutes at room temperature. Following binding of nuclei to beads, all further processing was done on ice. Bead bound nuclei from each nuclei preparation was washed 2x with 5 mL wash buffer (20 mM HEPES NaOH pH 7.5, 150 mM NaCl, 500 μM Spermidine (Sigma #S0266), and 0.5% Ultrapure BSA (Invitrogen #AM2618) with 1 tablet of Complete Protease Inhibitor Cocktail (Roche #11873580001) per 50 mL). After the second wash, nuclei bound beads were resuspended in 2.25 mL of wash buffer and 250 μL of nuclei bound beads (~200,000 nuclei) in wash buffer were added to individual microcentrifuge tubes corresponding to each antibody.

Supernatant was removed and beads were resuspended in 250 μL of antibody buffer (wash buffer + 0.05% Digitonin (Calbiochem #11024–24-1) + 2 mM EDTA) containing an individual antibody – anti-MeCP2 (rabbit monoclonal D4F3 Cell Signaling Technology #3456, clone D4F3, RRID: AB_2143894; 1:125 dilution), anti-Tri-Methyl-Histone H3 (Lys27) [H3K27me3] (rabbit monoclonal C36B11, Cell Signaling Technology #9733, RRID: AB_2616029; 1:125 dilution), anti-Acetyl-Histone H3 (Lys 27) [H3K27ac] (rabbit monoclonal D5E4, Cell Signaling Technology #8173, RRID: AB_10949503; 1:125 dilution), anti-Tri-Methyl-Histone H3 (Lys9) [H3K9me3] (rabbit polyclonal, abcam #ab8898, RRID: AB_306848; 1:100 dilution), anti-Acetyl-Histone H3 (Lys9) [H3K9ac] (rabbit monoclonal C5B11, Cell Signaling Technologies #9649, RRID: AB_823528; 1:125 dilution), anti-Di and Tri-Methyl-Histone H3 (Lys20) [H4K20me2/3] (mouse monoclonal 6F8-D9, abcam #ab78517, RRID: AB_1951279; 1:125 dilution), and rabbit IgG (Millipore #12–370, RRID: AB_145841; 1:125 dilution). Antibody buffer was added to nuclei bound beads during light vortexing (1100 rpm). Tubes were then placed at 4°C to rotate overnight. Following rotation, a quick spin on a microcentrifuge was performed to remove liquid from the cap and then washed 2x with 1 mL dig-wash buffer (wash buffer + 0.05% digitonin). Supernatant was removed and beads were resuspended in 100 μL 1x pAG-MNase (Epicypher #15–1016); 20x stock pAG-MNase) in dig-wash buffer and mixed with gentle flicking. Tubes were placed on nutator for 1hr at 4°C. Following incubation, samples were washed 2x with 1 mL dig-wash buffer.

To initiate cleavage and release of DNA bound fragments, each sample was resuspended in 150 μL of dig-wash buffer while gently vortexing. Samples were placed on ice in 4°C room for 10 minutes to equilibrate. To start digestion, while in 4°C room, 3 μL of 100 μM CaCl_2_ was added to each tube, quickly flicked, and immediately returned to ice. Following 45-minute incubation on ice, 150 μL 2x STOP (340 mM NaCl, 20 mM EDTA, 4 mM EGTA, 0.05% Digitonin, 100 μg/mL RNAse A (ThermoFisher Scientific #EN0531), 50 μg/mL Glycogen (ThermoFisher Scientific #10814010) and 0.75 ng E. coli spike-in / sample (Epicypher #18–1401) mixture was added to each sample. Tubes were then incubated at 37°C for 30 mins to digest RNA and release DNA. E.coli spike-in control was used for normalization of CUT&RUN signal due to differences in library amplification and/or sequencing. Supernatant was transferred to new tube and incubated with 1.5 μL 20% SDS and 5 μL 10 mg/mL Proteinase K while lightly shaking at 50°C for 1hr. DNA was then purified by phenol-chloroform extraction using Maxtract Tubes (129046, Qiagen) and pellet resuspended in 36.5 μL TE Buffer.

#### CUT&RUN Next Generation Sequencing Library Preparation

Library preparation was modified from protocols.IO (dx.doi.org/10.17504/protocols.io.bagaibse) utilizing reagents from the NEB Next II DNA Ultra Kit (New England Biolabs #E7645S) and Unique Combinatorial Dual index kit (New England Biolabs #E6440S, #E6442S, #E6444S) with modifications outlined below. Input DNA was quantified with Qubit, and 6ng of CUT&RUN DNA was used as input for the histone samples and 25 μl of CUT&RUN DNA was used for both MeCP2 and IgG samples. Volume of DNA was brought up to 25 μL and 1.5 μL End Prep Enzyme Mixture and 3.5 μL Reaction buffer were added and incubated at 20°C for 30 mins and 50°C for 60 mins. After end prep, 15 μL of NEB Next Ultra Ligation Mastermix, 0.5 μL Ligation Enhancer and 1.25 μL of Adapter (1.2 pmol adapter for histone modifications, 0.6 pmol adapter for MeCP2 and IgG) were added directly to the PCR tube, mixed by pipetting, and incubated for 15 mins at 20°C. Then, 1.5 μL of USER Enzyme is added to each tube. Finally, SPRI select beads (Beckman Coulter #B23318) were used at 1.6x ratio to remove excess adapter and eluted in 15 μL of TE buffer.

PCR amplification was performed using 13 μL of adaptor ligated fragments, 1 μL of Unique Combinatorial Dual Index (one index per sample), 1 μL of sterile water, and 15 μL 2x Q5 Master Mix. 14 cycles of PCR were performed with 10 seconds of denaturation at 98°C and 10 sec of annealing/extension at 65°C. Following PCR amplification, SPRI select beads were used for two-sided size selection; 0.65x right sided selection was performed first followed by 1.2x left sided size selection. Sample was eluted in 15 μl TE.

For quality control, each library size distribution was determined by Agilent Tapestation HS DNA 1000 (Agilent Technologies #5067) and concentration was determined by KAPA PCR (Roche #07960140001). Libraries were pooled together at equimolar concentrations. Each library was sequenced for approximately 40 million paired end reads of 150bp in length on a Novaseq S4 flow cell.

### CUT&RUN analysis

#### CUT&RUN Sequence Alignment

Our CUT&RUN data analysis pipeline was adapted from CUT&RUN Tools^81^. Raw Fastq files were appended together using Linux cat function. Adapter sequences were removed from sequence reads using Trimmomatic v.0.36 (2:15:4:4:true LEADING:20 TRAILING:20 SLIDINGWINDOW:4:15 MINLEN:25) from the Truseq3.PE.fa adapter library and kseq^64,81^. Confirmation of adapter removal and read quality was performed with fastqc (v0.11.8). Alignment was performed with bowtie2–2.3.4.1 (--dovetail --phred33) to both mm10 (GENCODE GRCm38p6 primary assembly version 18) and the spike-in Ecoli K12 Genomes (GCF_000005845.2_ASM584v2). Bedtools (v2.29.1) was used to process BAM files to BED files, remove blacklist (mm9 blacklist lifted over to mm10 and combined with mm10 blacklist downloaded with CUT&RUNTools ^81^ and to generate bedgraphs^77^. Each sample was normalized to internal Ecoli spike-in utilizing spike_in_calibration.sh as described previously (Meers et al., 2019). Both spike-in normalized bedgraphs for each sample and merged bedgraphs were converted to bigwigs using UCSC bedgraph to bigwig. Spike-in normalized bigwigs from each genotype were merged using deeptools bigwigCompare and averaged for summary figures. Integrated Genome Viewer (IGV) v2.11.1 was used to examine spike-in normalized bigwig tracks at individual loci^83^.

#### Binning and quantification of CUT&RUN signals

We extracted reads per genomic region using the bedtools function multicov for each BAM file generated from a CUT&RUN profile as previously described using the GENCODE GRCm38 release 23 coordinates^39,84^. Regions for quantification were defined as one meta gene per transcript encompassing: 1) the gene body between transcriptional start and end sites and 2) the promoter as defined as 2 kb upstream of the transcriptional start site. Counts were analyzed using DESeq2 package v.1.34.0 using the E-coli spike in control count as a normalization factor and cooksCutoff set to FALSE. Each mark was analyzed separately, with all time points analyzed together. Each mark at a specific time point was analyzed as a separate contrast. The log_2_ fold-change of aKO sample over control was calculated. The fold-changes per mark per time point were grouped by cluster identity and compared using Kruskal-Wallis test with Dunn’s post hoc test using the kwAllPairsDunnTest function in the PMCMRplus v.1.9.6 package in R. Robustness of global trends were assessed using bootstrapped subsamples of random background genes (see below).

#### Decay constant quantification

To assess the rate of MeCP2 depletion locally in the genome, we first used the DESeq2 normalized MeCP2 occupancy counts from the CUT&RUN signal extracted from the genomic regions of coding genes detected by RNA-seq (see above). We then normalized the aKO counts to the control counts at each respective time point, setting the control signal to 1 at each time point. We then fit a simple exponential decay *y* = e^t*x*^ for each gene, with the t parameter representing the decay constant. The decay constants were grouped by cluster identity and compared using Kruskal-Wallis test with Dunn’s post hoc test using the kwAllPairsDunnTest function in the PMCMRplus v.1.9.6 package in R. Robustness of global trends were assessed using bootstrapped subsamples of random background genes (see below).

#### Repeat region analysis

Raw CUT&RUN reads were adapter and quality trimmed using Trimgalore (https://github.com/FelixKrueger/TrimGalore). Trimmed reads were aligned separately to the mouse (mm10) and E. coli (U00096.3) reference genomes with Bowtie2 (bowtie2 -q -R 3 -N 1 -L 20 -i S,1,0.50 --end-to-end --dovetail --no-mixed -X 2000)^85^. Multimapping reads were randomly assigned. Duplicate reads were identified and removed using Picard. Reads which mapped to the mitochondrial genome were removed with Samtools samtools idxstats $sample.sorted.bam | cut -f 1 | grep -v chrM | xargs samtools view -b $sample.sorted.bam)^76^. Deduplicated bam files were then down-sampled according to the lowest E. coli spike-in reads per CUT&RUN antibody such that bams should be comparable across time points. Using the dfam’s non-redundant repetitive element annotations for mm10 (mm10.nrph.hitz.gz), we generated a bedfile of the locations of each family of mouse repetitive elements^86^. To determine chromatin compartments, peaks for H3K27ac and H3K9me3 were identified using the MACS2 algorithm v2.1.2 using IgG as input^87^. H3K27ac peaks were identified using the narrow peak caller, while H3K9me3 peaks were called using the broad peak caller using the –broad setting. Peaks were merged from all vehicle treated samples across time points. The “all” genomic compartment represents all loci of a family that are first in a primary assembly of a chromosome and had a non-zero number of alignments in both the control and aKO samples. The “heterochromatin” genomic compartment is a subset of these “all” loci that intersect with an H3K9me3 peak, while the “euchromatin” genomic compartment is a subset of these “all” loci that intersect with an H3K27ac peak. Reads from each repeat family were then counted at each of these compartment bins using featureCounts^88^. Reads which mapped to partial assemblies were removed. The counts from individual loci were then aggregated for each subfamily using grep and awk and subfamilies were further grouped together as appropriate. A log_2_ fold change (Tamoxifen/Vehicle) of the median value of each family was then calculated and plotted using pheatmap in R. In order to perform statistical assessments of changes per family, a Wilcoxon Rank Sum (Mann–Whitney U) test was performed in R comparing Tamoxifen versus Vehicle reads per kilobase at a family’s loci. Raw p-values were then adjusted using Benjamini Hochberg correction.

### Bootstrap subsample analyses

To assess if characteristics of persistently dysregulated genes were different than background genes, we performed random subsampling. We first randomly pulled a subset of genes of equal number to the average of the up- and down-regulated cluster from the non-persistently dysregulated genes. We then sampled a second independent, non-overlapping subset of genes of equal size. For each variable compared, we performed a Kruskal-Wallis test with Dunn’s post-hoc comparisons using the kwAllPairsDunnTest function in the PMCMRplus v.1.9.6 package in R and stored the *p*-adjusted value per subsample. Distributions of *p*-adjusted values are displayed as boxplots.

### Western Blot protein measurements

Tissues were homogenized in lysis buffer (10 mM HEPES pH 7.9, 3 mM MgCl_2_, 5 mM KCl, 140 mM NaCl, 0.1 mM EDTA, 0.5 mM DDT, 0.5% NP-40, 2% SDS). To lysis buffer, 1x protease and 1x phosphatase inhibitors were added fresh (Xpert Phosphatase Inhibitor Cocktail GenDEPOT #P3200–001 and Xpert Protease Inhibitor Cocktail GenDEPOT #P3100–001). One hippocampus was homogenized in 700 μL complete lysis buffer using a motorized pestle on ice. Tissue was further homogenized by passing lysate through a 27-gauge needle eight times. Lysates were sonicated using a Bioruptor Pico (diagenode #B01060010) for 10 cycles of 30 seconds sonication, 30 seconds no sonication. After sonication, lysates were rotated for 20 minutes at room temperature and then spun down at maximum speed on a benchtop centrifuge for 15 minutes at room temperature. Supernatants were transferred to fresh tubes and stored at −80°C until sample preparation. Protein concentration was assessed using the Peirce BCA Protein Assay (ThermoFisher #23225) according to manufacturer’s protocol. 15 μg of total protein was mixed with 1x NuPAGE LDS sample buffer (ThermoFisher #NP0007) with 1x NuPAGE Sample Reducing Agent (ThermoFisher #NP0004) and boiled at 95°C for 5 minutes. Prepared samples were run on a NuPAGE 4–12% Bis-Tris gradient gel with MOPS SDS running buffer (Boston BioProducts #BP-178). Separated proteins were transferred to a 0.2 μm pore-size nitrocellulose membrane (Bio-Rad #1704271) using the Trans-Blot Turbo Transfer System (Bio-Rad #1704150EDU) following manufacturer’s recommendations of 2.5 A and 25 V for seven minutes. The membranes were blocked in 0.5x Odyssey Blocking buffer (TBS; LiCOR #927–50000) for 1 hour at room temperature. Membranes were then probed with primary antibody diluted in 0.5x blocking buffer + 0.1% Tween-20. The following antibodies and dilutions were used: anti-MeCP2 (rabbit monoclonal D4F3, Cell Signaling Technology #3456, RRID:AB_2143849; 1:1000 dilution), anti-Vinculin (mouse monoclonal hVIN-1, MilliporeSigma #V9131, RRID: AB_477629; 1:20000 dilution), anti-GAPDH (mouse monocloncal 6C5, Advanced ImmunoChemical Inc. #2-RGM2, RRID: AB_2721282, 1:20000 dilution), anti-alpha tubulin (chicken polyclonal, abcam #ab89984, RRID: AB_10672056, 1:20000 dilution). Membranes were washed three times with TBS-T for 10 minutes and incubated in LiCOR secondary antibodies (1:20000 dilution) in 0.5x blocking buffer + 0.1% Tween-20 + 0.1% SDS for 1 hour at room temperature. Membranes were washed three times with TBS-T for 10 minutes and one time with TBS for 10 minutes. Membranes were then imaged on a LiCOR CLx imager. Band intensities were quantified using the Analyze Gel commands in Fiji. MeCP2 intensities were normalized to the average intensity of the three loading controls. Data are presented as mean ± sem in figures. Raw scans and quantification are uploaded to a Zenodo repository for access: https://zenodo.org/records/13785439?token=eyJhbGciOiJIUzUxMiIsImlhdCI6MTcwMzI4MDkyNywiZXhwIjoxNzM1Njg5NTk5fQ.eyJpZCI6IjQ5MTIyYjVmLWI5NmQtNDg3Ni1hMmQzLWQzY2U3OGE4Y2MwYiIsImRhdGEiOnt9LCJyYW5kb20iOiJlNTI3MmI2MGMxMjM4OWI2NDgyZGQ1ZmZhYjY2MTY3MiJ9.ioeB4VkV5pqnun3nInVEDfD_qIbp6e4jfIXPo08dvZcVTyOGdymWhn5417APMDWy4LFRty9ZkjjuOLcm8jEpiQ

### Behavioral assays

All behavioral assays were performed during the light period. Mice were habituated to the test room for at least 30 minutes before each test. Mice were given at least one day to recover between different assays. All testing, data acquisition, and analyses were carried out by an individual blinded to the genotype. The open field assay was performed first, followed by the fear conditioning assay at the specific time point after treatment. Each time point is a separate treated cohort and tested as approximately similar time points. Outlier analysis was not performed on the data points, and no data points were removed from any assays.

#### Open Field Assay

The lighting in the test room was set to 150 lux, and the background noise level was set to 62 dB with a white noise generator. After habituation, mice were placed in an open plexiglass area (40 × 40 × 30 cm), and their movement and behavior were tracked by laser photobeam breaks. Mice were allowed to freely move for 30 minutes. The total distance traveled, horizontal or vertical laser beam breaks (activity counts), entries into the center 10 × 10 cm, and time in the center 10 × 10 cm were recorded and tabulated by the AccuScan Fusion software (Omnitech Electronics Inc).

#### Fear Conditioning

Animals were habituated in an adjacent room to the testing room with a light level of 150 lux and noise level of 62 dB using a white noise generator. On the training day, mice were brought to the testing groom (ambient light, no noise) and placed into a chamber containing a grid floor that can deliver an electric shock with one mouse per chamber (Med Associates Inc). The chamber was housed within a sound-attenuating box with a digital camera, loudspeaker, and a light. The training paradigm consisted of 2 minutes of no noise or shock, then a tone for 30 seconds (5 kHz, 85 dB), ending with a foot shock for 2 seconds (1 mA). The tone and shock pairing was repeated after 2 minutes of no noise or shock. The mice were returned to their home cages after training. On the testing day, occurring 24 hours later, two tests are performed – context and cued learning. For the context test, the mice were placed in the exact same chamber with no noise or shock for 5 minutes. After one-hour post-context test, the cued learning test was performed. For the cued learning test, the mice were placed in a novel environment for 6 minutes. The first 3 minutes with no noise or shock, and the last 3 minutes with tone only (5 kHz, 85 dB). Movement was recorded on video and freezing, as defined by absence of all movement except respiration, was quantified using the FreezeFrame software (ActiMetrics) using a bout duration of 1 second and movement threshold of 10.

### Hippocampal slice preparation and long-term potentiation electrophysiology

Mice were anaesthetized by isoflurane inhalation and decapitated. The brain was isolated and immersed in chilled (2–5°C) cutting solution containing 220 mM sucrose, 2.5 mM KCl, 0.5 mM CaCl_2_, 7 mM MgCl_2_, 1.25 mM NaH_2_PO_4_, 25 mM NaHCO_3_, and 7 mM D-glucose. Transverse hippocampal slices (400 μm) were prepared with a vibratome (Leica Microsystems Inc., Buffalo Grove, IL). The slices were incubated at 34°C for 60 minutes in artificial cerebrospinal fluid (ACSF) containing 125 mM NaCl, 2.5 mM KCl, 2 mM CaCl_2_, 1 mM MgCl_2_, 1.25 mM NaH_2_PO_4_, 25 mM NaHCO_3_, and 14.83 mM D-glucose, and then transferred to room temperature.

Extracellular stimuli were administered along the Schaffer collaterals using a bipolar tungsten stimulation electrode (WPI), and field excitatory postsynaptic potentials (fEPSPs) were recorded in stratum radiatum. The recording pipettes were filled with 2 M NaCl (1–2 MΩ) and their distance to the stimulating electrode were kept constant (~300 μm). Input/output (I/O) curves were generated using incremental stimulus intensities and were used to assess baseline synaptic transmission. All subsequent stimuli were set to an intensity that evoked a 30–40% of the maximum fEPSP slope. Paired pulse facilitation was assessed via systematic stimuli with different interval (25, 50, 100, 200, and 400 ms). The paired pulse ratio was obtained by dividing the rising slope of the second fEPSP with the rising slope of the first fEPSP. For LTP experiments, stable baseline fEPSPs were recorded for 20 min, and then LTP was induced by 2 trains of high-frequency stimulation (HFS, 100 Hz for 1 s) with 20 s intertrain interval. The magnitude of potentiation was determined by measuring the rising slope of the fEPSP. For electrophysiologic measurements, all results are expressed as mean ± SEM. fEPSP slope was normalized to the baseline. LTP (%) were calculated as follows: 100 × [mean fEPSP slope during the final 10 min of recording/ mean baseline fEPSP slope].

The recordings were performed using Multiclamp 700B amplifiers (Molecular Devices, Union City, CA). Data acquisition and analysis were performed using digitizer DigiData 1440A and analysis software pClamp 11 (Molecular Devices). Signals were filtered at 2 kHz and sampled at 10 kHz. All recordings were performed at 30 ± 1°C by using an automatic temperature controller (Warner Instrument, Hamden, CT).

### Statistical methods

Statistical methods for bioinformatic analyses are stated in the Methods section for each technique and analysis respectively. Statistical analyses for biochemical, behavioral, and electrophysiologic assays are stated in the Figure captions for which these assays are described. Values of symbols denoting *p*-value significance are also stated in the Figure captions.

## Supplementary Material

1

2 Table S1. Raw and DESeq2 normalized RNA-sequencing counts, related to Figure 2. This file contains a spread sheet with multiple tables containing the raw and normalized RNA-sequencing counts. Excel file containing additional data too large to fit in a PDF.

3 Table S2. DESeq2 data frame from treatment controls, related to Figure 2. This file contains a spread sheet with multiple tables containing the raw and normalized RNA-sequencing counts from tamoxifen-treated wild-type, Flox-only, Cre-only samples relative to Flox; Cre – vehicle treated. Excel file containing additional data too large to fit in a PDF.

4 Table S3. Gene ontology analysis of gene programs induced by loss of MeCP2 per time point, related to Figure 2. GO terms and associated enrichment for the up- and down-regulated genes per time point. Excel file containing additional data too large to fit in a PDF.

5 Table S4. DESeq2 data frame from adult knockout; Fisher p-value, related to Figures 2 and 4. This file contains a spread sheet with multiple tables containing the raw and normalized RNA-sequencing counts from tamoxifen-treated Flox; Cre samples relative to Flox; Cre – vehicle treated along with Fisher p-value calculations across time. Excel file containing additional data too large to fit in a PDF.

6 Table S5. DESeq2 data from MeCP2 CUT&RUN, related to Figure 5. This file contains DESeq2 analysis of MeCP2 binding per meta-gene of detected genes. Excel file containing additional data too large to fit in a PDF.

7 Table S6. DESeq2 data from Histone CUT&RUN, related to Figure 6. This file contains DESeq2 analysis of histone binding per meta-gene of detected genes. Each tab is a separate chromatin mark. Excel file containing additional data too large to fit in a PDF.

8 Table S7. Quantification of epigenome binding at repeat regions by compartment, related to Figure 6, Supplemental Figure S10, and STAR Methods. This file contains analysis of histone binding per repeat region type in chromatin regions. Each tab is a separate chromatin mark. Excel file containing additional data too large to fit in a PDF.

## Figures and Tables

**Key resources table:** 

REAGENT or RESOURCE	SOURCE	IDENTIFIER
Antibodies		
Rabbit monoclonal anti-MeCP2 (clone D4F3)	Cell Signaling Technology	#3456; RRID: AB_2143894
Rabbit monoclonal anti-Tri-Methyl-Histone H3 (Lys27) [H3K27me3] (clone C36B11)	Cell Signaling Technology	#9733; RRID: AB_2616029
Rabbit monoclonal anti-Acetyl-Histone H3 (Lys27) [H3K27ac] (clone D5E4)	Cell Signaling Technology	#8173, RRID: AB_10949503
Rabbit polyclonal anti-Tri-Methyl-Histone H3 (Lys9) [H3K9me3]	Abcam	#ab8898; RRID: AB_306848
Rabbit monoclonal anti-Acetyl-Histone H3 (Lys9) [H3K9ac] (clone C5B11)	Cell Signaling Technology	#9649, RRID: AB_823528
Mouse monoclonal anti-Di and Tri-Methyl-Histone H3 (Lys20) [H4K20me2/3] (clone 6F8-D9)	Abcam	#ab78517, RRID: AB_1951279
Rabbit polyclonal IgG	Millipore	#12–370, RRID: AB_145841
Mouse monoclonal anti-Vinculin (clone hVIN-1)	MilliporeSigma	#V9131, RRID: AB_477629
Mouse monoclonal anti-GAPDH (clone 6C5)	Advanced ImmunoChemical Inc.	#2-RGM2, RRID: AB_2721282
Chicken polyclonal anti-alpha tubulin	Abcam	#ab89984, RRID: AB_10672056
		
Bacterial and virus strains		
		
		
		
		
		
Biological samples		
		
		
		
		
		
Chemicals, peptides, and recombinant proteins		
Tamoxifen	Sigma-Aldrich	#T5648
Peanut oil	Sigma-Aldrich	#P2144
OptiPrep density gradient	Sigma-Aldrich	#D15556
Spermidine	Sigma-Aldrich	#S0266
Ultrapure BSA	Invitrogen	#AM2618
cOmplete protease inhibitor	Roche	#11873580001
Digitonin	Calbiochem	#11024–24–1
pAG-MNase	Epicypher	#15–1016
		
		
		
Critical commercial assays		
Qiagen miRNeasy Mini	Qiagen	#217004
Qiagen RNase free DNase	Qiagen	#79254
NEB Next II DNA Ultra Kit	New England Biolabs	#E7645S
Unique Combinatorial Dual index kit	New England Biolabs	#E6440S, #E6442S, #E6444S
		
		
		
		
Deposited data		
*Mecp2* Adult Knockout RNA-sequencing (raw and processed)	This study	GEO: GSE246119
*Mecp2* Adult Knockout CUT&RUN (raw and processed)	This study	GEO: GSE246664
*Mecp2*-G118E RNA-sequencing (raw and processed)	This study	GEO: GSE277077
*Mecp2*-null Cortex RNA-sequencing	Zhou et al.^36^	GEO: GSE179229
*Mecp2*-null Cortex RNA-sequencing	Clemens et al.^10^	GEO: GSE123372
*Mecp2*-null Cortex RNA-sequencing	Boxer et al.^9^	GEO: GSE128178
*Mecp2*-R306C Cortex RNA-sequencing	Boxer et al.^9^	GEO: GSE128178
*Mecp2*-null Hypothalamus RNA-sequencing	Chen et al.^20^	GEO: GSE66870
*Mecp2*-null Cerebellum RNA-sequencing	Raman et al.^37^	GEO: GSE105045
Reduced representation bisulfite sequencing of hippocampus	Reizel et al.^41^	GEO: GSE85251
Hippocampal RNA-seq used in deconvolution	Habib et al.^67^	GEO: GSE143758
Hippocampal RNA-seq used in deconvolution	Methi et al.^68^	GEO: GSE234278
		
Experimental models: Cell lines		
		
		
		
		
		
Experimental models: Organisms/strains		
*B6.129S4(C)-Mecp2^tm1Jae^/Mmucd*	MMRCC	MMRCC# 011918-UCD,; RRID:MMRRC 011918-UCD
B6.*Cg-Ndor1^Tg(UBC-cre/ERT2)1Ejb^/1J*	The Jackson Labs	Jax #007001; RRID: IMSR_JAX:007001
*Mecp2*-G118E	Zhou et al.^36^	N/A
C57BL6/J	The Jackson Labs	Jax #000664; IMSR_JAX:000664
		
		
Oligonucleotides		
*Ppia* qPCR forward primer:	This paper	N/A
*Ppia* qPCR reverse primer	This paper	N/A
*Mecp2* qPCR forward primer:	This paper	N/A
*Mecp2* qPCR reverse primer:	This paper	N/A
		
Recombinant DNA		
		
		
		
		
		
Software and algorithms		
STAR aligner	Dobin et al.^65^	https://github.com/alexdobin/STAR
DESeq2	Love et al.^66^	https://bioconductor.org/packages/release/bioc/html/DESeq2.html
CIBERSORTx	Newman et al.^71^	https://cibersortx.stanford.edu/
Bismark	Krueger and Andrews^75^	https://github.com/FelixKrueger/Bismark
samtools	Li et al.^76^	https://www.htslib.org/
Trimmomatic	Bolger et al.^64^	http://www.usadellab.org/cms/?page=trimmomatic
Trimgalore	N/A	https://github.com/FelixKrueger/TrimGalore
bedtools	Quinlan and Hall^77^	https://bedtools.readthedocs.io/en/latest/
MACS2	Zhang et al.^87^	https://pypi.org/project/MACS2/
edgeR	Robinson et al.^72^	https://bioconductor.org/packages/release/bioc/html/edgeR.html
deepTools	Ramirez et al.^79^	https://deeptools.readthedocs.io/en/develop/
		
Other		
Concavalin A magnetic beads	Bangs Laboratories Inc.	#BP531
SPRI select beads	Beckman Coulter	#B23318
E. coli spike in	Epicypher	#18–1401

		
		
		
		
		

**Table:** LIFE SCIENCES

REAGENT or RESOURCE	SOURCE	IDENTIFIER
Antibodies		
Rabbit monoclonal anti-Snail	Cell Signaling Technology	Cat#3879S; RRID: AB_2255011
Mouse monoclonal anti-Tubulin (clone DM1A)	Sigma-Aldrich	Cat#T9026; RRID: AB_477593
Rabbit polyclonal anti-BMAL1	This paper	N/A
Bacterial and virus strains		
pAAV-hSyn-DIO-hM3D(Gq)-mCherry	Krashes et al.^1^	Addgene AAV5; 44361-AAV5
AAV5-EF1a-DIO-hChR2(H134R)-EYFP	Hope Center Viral Vectors Core	N/A
Cowpox virus Brighton Red	BEI Resources	NR-88
Zika-SMGC-1, GENBANK: KX266255	Isolated from patient (Wang et al.^2^)	N/A
*Staphylococcus aureus*	ATCC	ATCC 29213
*Streptococcus pyogenes*: M1 serotype strain: strain SF370; M1 GAS	ATCC	ATCC 700294
Biological samples		
Healthy adult BA9 brain tissue	University of Maryland Brain & Tissue Bank; http://medschool.umaryland.edu/btbank/	Cat#UMB1455
Human hippocampal brain blocks	New York Brain Bank	http://nybb.hs.columbia.edu/
Patient-derived xenografts (PDX)	Children’s Oncology Group Cell Culture and Xenograft Repository	http://cogcell.org/
Chemicals, peptides, and recombinant proteins		
MK-2206 AKT inhibitor	Selleck Chemicals	S1078; CAS: 1032350–13–2
SB-505124	Sigma-Aldrich	S4696; CAS: 694433–59–5 (free base)
Picrotoxin	Sigma-Aldrich	P1675; CAS: 124–87–8
Human TGF-β	R&D	240-B; GenPept: P01137
Activated S6K1	Millipore	Cat#14–486
GST-BMAL1	Novus	Cat#H00000406-P01
Critical commercial assays		
EasyTag EXPRESS 35S Protein Labeling Kit	PerkinElmer	NEG772014MC
CaspaseGlo 3/7	Promega	G8090
TruSeq ChIP Sample Prep Kit	Illumina	IP-202–1012
Deposited data		
Raw and analyzed data	This paper	GEO: GSE63473
B-RAF RBD (apo) structure	This paper	PDB: 5J17
Human reference genome NCBI build 37, GRCh37	Genome Reference Consortium	http://www.ncbi.nlm.nih.gov/projects/genome/assembly/grc/human/
Nanog STILT inference	This paper; Mendeley Data	http://dx.doi.org/10.17632/wx6s4mj7s8.2
Affinity-based mass spectrometry performed with 57 genes	This paper; Mendeley Data	Table S8; http://dx.doi.org/10.17632/5hvpvspw82.1
Experimental models: Cell lines		
Hamster: CHO cells	ATCC	CRL-11268
*D. melanogaster:* Cell line S2: S2-DRSC	Laboratory of Norbert Perrimon	FlyBase: FBtc0000181
Human: Passage 40 H9 ES cells	MSKCC stem cell core facility	N/A
Human: HUES 8 hESC line (NIH approval number NIHhESC-09–0021)	HSCI iPS Core	hES Cell Line: HUES-8
Experimental models: Organisms/strains		
*C. elegans:* Strain BC4011: srl-1 (s2500) II; dpy-18(e364) III; unc-46(e177)rol-3(s1040) V.	Caenorhabditis Genetics Center	WB Strain: BC4011; WormBase: WBVar00241916
*D. melanogaster:* RNAi of Sxl: y[1] sc[*] v[1]; P{TRiP.HMS00609}attP2	Bloomington Drosophila Stock Center	BDSC.34393; FlyBase: FBtp0064874
*S. cerevisiae:* Strain background: W303	ATCC	ATTC: 208353
Mouse: R6/2: B6CBA-Tg(HDexon1)62Gpb/3J	The Jackson Laboratory	JAX: 006494
Mouse: OXTRfl/fl: B6.129(SJL)-Oxtr^tm1.1Wsy^/J	The Jackson Laboratory	RRID: IMSR_JAX:008471
Zebrafish: Tg(Shha:GFP)t10: t10Tg	Neumann and Nuesslein-Volhard^3^	ZFIN: ZDB-GENO-060207–1
*Arabidopsis:* 35S::PIF4-YFP, BZR1-CFP	Wang et al.^4^	N/A
*Arabidopsis:* JYB1021.2: pS24(AT5G58010)::cS24:GFP(-G):NOS #1	NASC	NASC ID: N70450
Oligonucleotides		
siRNA targeting sequence: PIP5K I alpha #1: ACACAGUACUCAGUUGAUA	This paper	N/A
Primers for XX, see Table SX	This paper	N/A
Primer: GFP/YFP/CFP Forward: GCACGACTTCTTCAAGTCCGCCATGCC	This paper	N/A
Morpholino: MO-pax2a GGTCTGCTTTGCAGTGAATATCCAT	Gene Tools	ZFIN: ZDB-MRPHLNO-061106–5
ACTB (hs01060665_g1)	Life Technologies	Cat#4331182
RNA sequence: hnRNPA1 ligand: UAGGGACUUAGGGUUCUCUCUAGGGACUUAGGGUUCUCUCUAGGGA	This paper	N/A
Recombinant DNA		
pLVX-Tight-Puro (TetOn)	Clonetech	Cat#632162
Plasmid: GFP-Nito	This paper	N/A
cDNA GH111110	Drosophila Genomics Resource Center	DGRC:5666; FlyBase:FBcl0130415
AAV2/1-hsyn-GCaMP6- WPRE	Chen et al.^5^	N/A
Mouse raptor: pLKO mouse shRNA 1 raptor	Thoreen et al.^6^	Addgene Plasmid #21339
Software and algorithms		
ImageJ	Schneider et al.^7^	https://imagej.nih.gov/ij/
Bowtie2	Langmead and Salzberg^8^	http://bowtie-bio.sourceforge.net/bowtie2/index.shtml
Samtools	Li et al.^9^	http://samtools.sourceforge.net/
Weighted Maximal Information Component Analysis v0.9	Rau et al.^10^	https://github.com/ChristophRau/wMICA
ICS algorithm	This paper; Mendeley Data	http://dx.doi.org/10.17632/5hvpvspw82.1
Other		
Sequence data, analyses, and resources related to the ultra-deep sequencing of the AML31 tumor, relapse, and matched normal	This paper	http://aml31.genome.wustl.edu
Resource website for the AML31 publication	This paper	https://github.com/chrisamiller/aml31SuppSite

**Table:** PHYSICAL SCIENCES

REAGENT or RESOURCE	SOURCE	IDENTIFIER
Chemicals, peptides, and recombinant proteins		
QD605 streptavidin conjugated quantum dot	Thermo Fisher Scientific	Cat#Q10101MP
Platinum black	Sigma-Aldrich	Cat#205915
Sodium formate BioUltra, ≥99.0% (NT)	Sigma-Aldrich	Cat#71359
Chloramphenicol	Sigma-Aldrich	Cat#C0378
Carbon dioxide (^13^C, 99%) (<2% ^18^O)	Cambridge Isotope Laboratories	CLM-185–5
Poly(vinylidene fluoride-co-hexafluoropropylene)	Sigma-Aldrich	427179
PTFE Hydrophilic Membrane Filters, 0.22 μm, 90 mm	Scientificfilters.com/TischScientific	SF13842
Critical commercial assays		
Folic Acid (FA) ELISA kit	Alpha Diagnostic International	Cat# 0365–0B9
TMT10plex Isobaric Label Reagent Set	Thermo Fisher	A37725
Surface Plasmon Resonance CM5 kit	GE Healthcare	Cat#29104988
NanoBRET Target Engagement K-5 kit	Promega	Cat#N2500
Deposited data		
B-RAF RBD (apo) structure	This paper	PDB: 5J17
Structure of compound 5	This paper; Cambridge Crystallographic Data Center	CCDC: 2016466
Code for constraints-based modeling and analysis of autotrophic *E. coli*	This paper	https://gitlab.com/elad.noor/sloppy/tree/master/rubisco
Software and algorithms		
Gaussian09	Frish et al.^1^	https://gaussian.com
Python version 2.7	Python Software Foundation	https://www.python.org
ChemDraw Professional 18.0	PerkinElmer	https://www.perkinelmer.com/category/chemdraw
Weighted Maximal Information Component Analysis v0.9	Rau et al.^2^	https://github.com/ChristophRau/wMICA
Other		
DASGIP MX4/4 Gas Mixing Module for 4 Vessels with a Mass Flow Controller	Eppendorf	Cat#76DGMX44
Agilent 1200 series HPLC	Agilent Technologies	https://www.agilent.com/en/products/liquid-chromatography
PHI Quantera II XPS	ULVAC-PHI, Inc.	https://www.ulvac-phi.com/en/products/xps/phi-quantera-ii/

## Data Availability

RNA-sequencing and CUT&RUN profiles generated in this study are deposited in the Gene Expression Omnibus (GSE246119 – RNA-seq and GSE246664 – CUT&RUN). Previously published RNA-sequencing studies from *Mecp2*-null or *Mecp2*-R306C animals are available on the Gene Expression Omnibus (GSE66870, GSE105045, GSE128178, GSE123372, GSE179229). Previously published methylation profile from the hippocampus is available on the Gene Expression Omnibus (GSE85251). Raw western blot images are uploaded to a Zenodo repository: https://zenodo.org/uploads/13785439. This study does not report any original code and the utilized code to generate the analyses in this manuscript is available from the lead contact upon request. Any additional information required to reanalyze the data reported in this manuscript is available from the lead contact upon request.

## HIGHLIGHTS

- Deletion of *Mecp2* in adult mice triggers global transcriptional dysregulation
- MeCP2 acutely regulates a core set of transcripts
- Acute loss of MeCP2 triggers a subtle dysregulation in the chromatin landscape
- Molecular deficits precede phenotypic deficits upon adult loss of MeCP2
