# Supplementary material for: Acute MeCP2 loss in adult mice reveals transcriptional and chromatin changes that precede neurological dysfunction and inform pathogenic cascade
Source: Neuron. Author manuscript; Available in PMC 2025 Feb 7. (PMC11802321; doi:10.1016/j.neuron.2024.11.006)
Supplement: 1 [file NIHMS2040931-supplement-1.pdf]

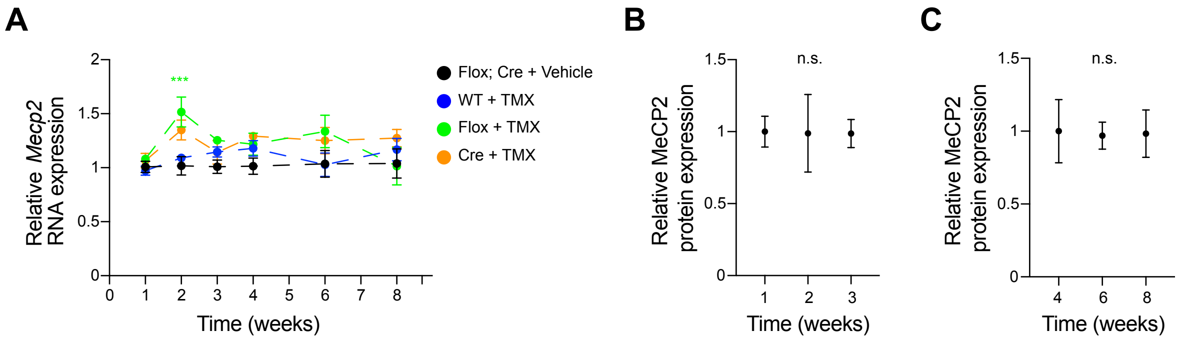

Figure S1. Related to Figure 1. *Mecp2* expression and MeCP2 levels are stable over time in adult mice. (A) Quantification of *Mecp2* RNA expression in the hippocampus after tamoxifen treatment as measured by qPCR. Tamoxifen treated wild-type (WT), Flox-only (Flox), and Cre-only (Cre) animals are referred to as treatment controls. Data are normalized to the control (Flox; Cre + vehicle) *Mecp2* expression per time point. (B,C) Quantification of MeCP2 protein levels in the hippocampi of control animals over 1-3 weeks (B) or 4-8 weeks (C) after vehicle injection. Quantification of western blot data was performed on samples on the same membrane. Data are shown as mean ± sem from n = 3-4 biological replicates. Data were analyzed by two-way ANOVA (A) or one-way ANOVA (B,C) and post-hoc multiple comparisons, with (***) $P < 0.001$. Raw blots and analyses are included in the Zenodo repository linked in the Methods.

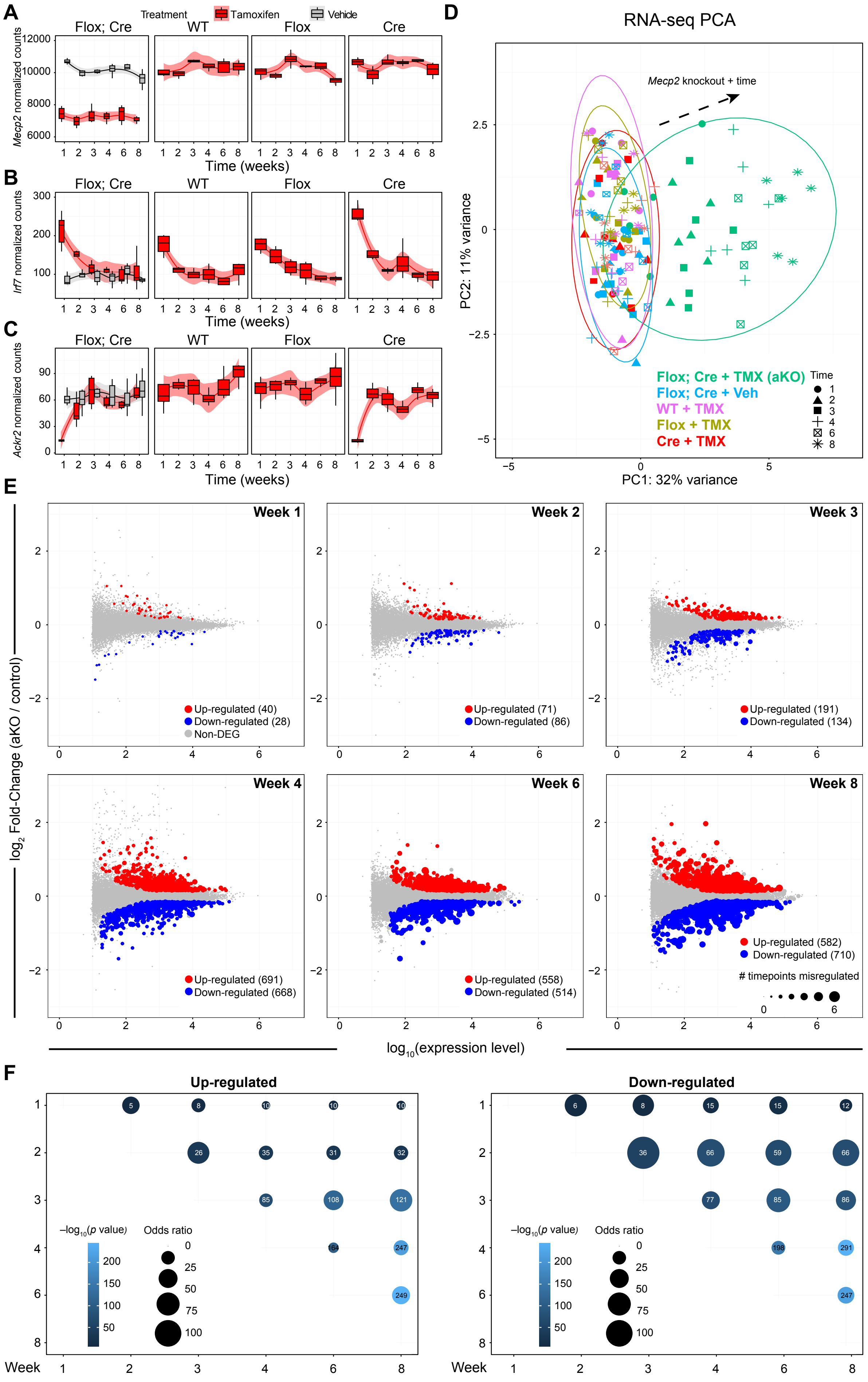

A
Treatment
Tamoxifen
Vehicle
Flox; Cre
WT
Flox
Cre
Mecp2 normalized counts
Time (weeks)
B
Irf7 normalized counts
C
Ackr2 normalized counts
D
RNA-seq PCA
Mecp2 knockout + time
PC2: 11% variance
PC1: 32% variance
Flox; Cre + TMX (aKO)
Flox; Cre + Veh
WT + TMX
Flox + TMX
Cre + TMX
Time
E
Week 1
Up-regulated (40)
Down-regulated (28)
Non-DEG
Week 2
Up-regulated (71)
Down-regulated (86)
Week 3
Up-regulated (191)
Down-regulated (134)
Week 4
Up-regulated (691)
Down-regulated (668)
Week 6
Up-regulated (558)
Down-regulated (514)
Week 8
Up-regulated (582)
Down-regulated (710)
timepoints misregulated
log2 Fold-Change (aKO / control)
log10(expression level)
F
Up-regulated
Down-regulated
−log10(p value)
Odds ratio
Week

Figure S2. Related to Figure 2. MeCP2-dependent gene dysregulation over time after adult knockout. Schematic of sample collection is shown in Figure 2. (A) Normalized expression of *Mecp2* over time using RNA-sequencing. (B) Normalized expression of *Irf7* over time using RNA-sequencing. Note the increased expression at week 1 in *Mecp2* adult knockout (aKO) and treatment controls compared to control, vehicle treated samples. Line and ribbon represent a LOESS fit to the expression values. (C) Normalized *Ackr2* expression over time using RNA-sequencing. Note the decreased expression at week 1 of aKO and Cre+TMX samples compared to control, vehicle treated samples. (D) Principal component analysis of all the RNA-sequencing samples analyzed in this study. Sample are colored by genotype and the shape indicates the time point of sample collection. A summarizing oval is shown for each genotype (n = 3-6). (E) Comparison of expression level and $\log_2$ Fold-Change (aKO / control). Down-regulated genes are shown in blue, and up-regulated genes are shown in red ($p_{adj} < 0.05$ and | $\log_2$ Fold-Change (aKO / control) | > 0.15). The number of genes in each category are denoted in the lower right. The size of the data point corresponds to the number of time points a given gene was determined to be differentially expressed. (F) Overlap of differentially expressed genes throughout time. Each dot represents the pair-wise gene expression overlap between the time point on the x-axis and y-axis. Dot circle represents the odds ratio of overlap, while the color represents the $-\log_{10}$ p-value of overlap as calculated by Fisher's exact test. The total number of overlapped genes is displayed in the dot. Genes are split by directionality of regulation.

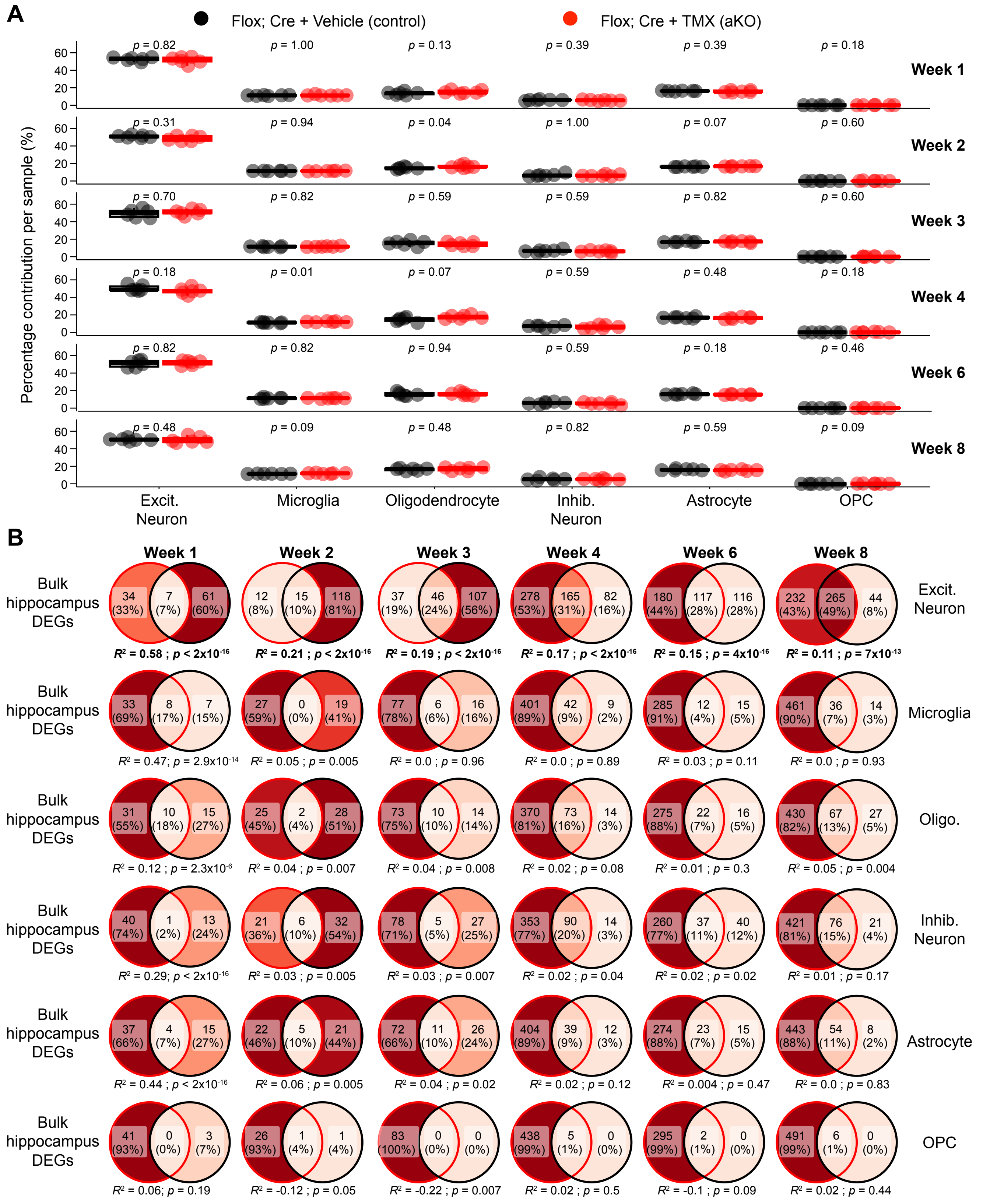

Figure S3. Related to Figure 2. Deconvolved cell-type specific contributions in bulk RNA-sequencing profiles generated in *Mecp2* adult knockout hippocampi. (A) Deconvolved cell type proportions to the overall gene expression signature between control and aKO hippocampi. Cell types were identified using markers derived from previously published single-nuclei RNA-sequencing datasets (see Methods). (B) Cell-

type specific gene expression was computed using the CIBERSORTx algorithm. Differential gene expression per cell population was computed using a Wilcoxon test between aKO and control samples. Venn Diagrams display the gene overlap of significant differentially expressed genes (DEGs) between the bulk expression and cell-type specific expression. The correlation of fold-changes between bulk expression and cell-type specific expression was quantified using Pearson correlation. The R^2 and p-value of correlation per comparison is denoted below the Venn Diagram. The highest significant correlation per cell type per time point is bolded. Cell type abbreviations: Excit. neuron - excitatory neurons; Inhib. neuron – inhibitory neuron; Oligo. – oligodendrocyte; OPC – oligodendrocyte precursor cell.

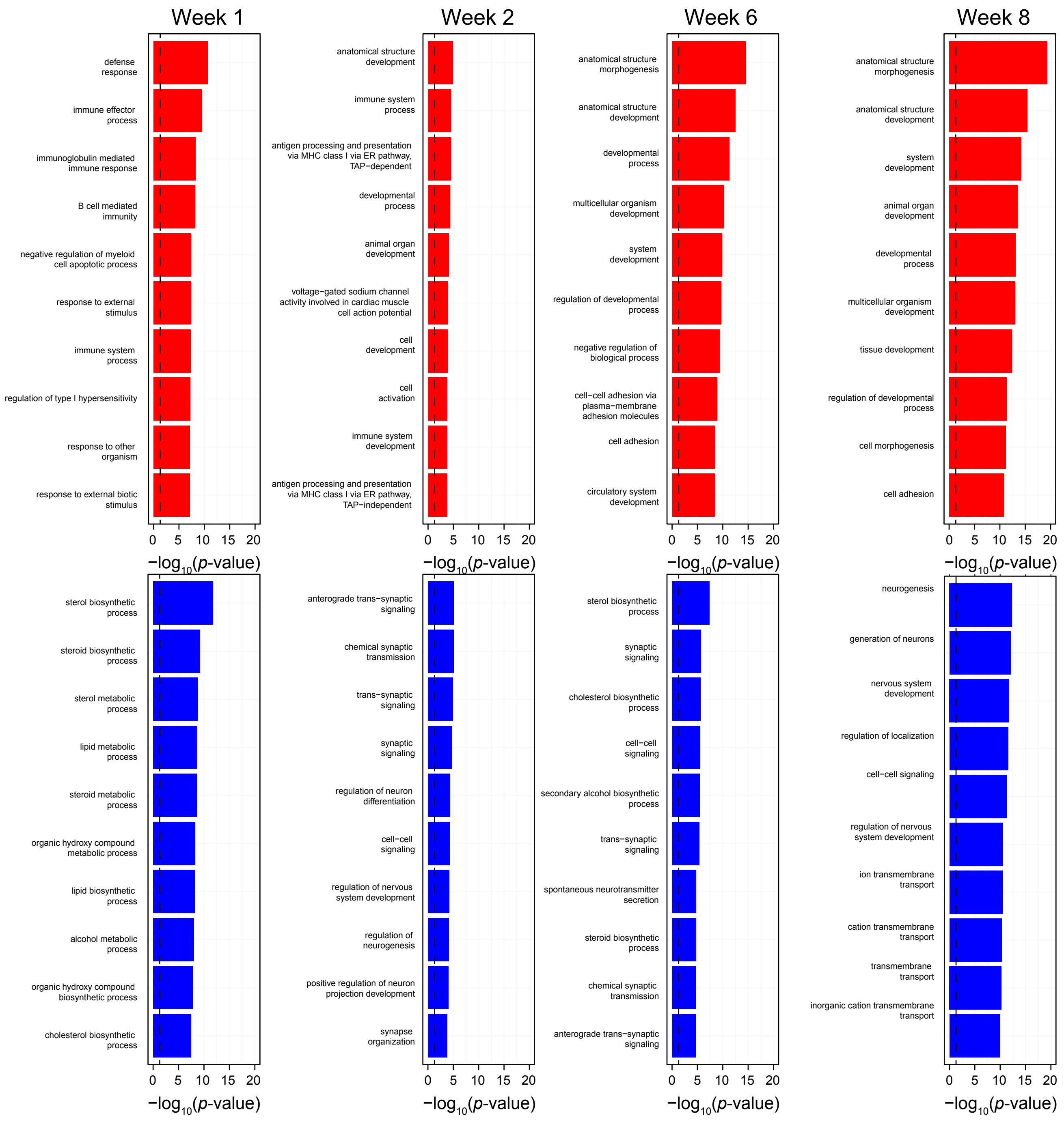

Figure S4. Related to Figure 2. Gene ontology analysis of gene programs dysregulated early or late after loss of MeCP2. Gene expression signatures that are up- (red) or down-(blue) regulated at early (one- and two-week, left) or late (six- and eight-week, right) time points were identified using gene ontology. The top 10 most significant programs per gene set are displayed in the bar graph. The x-axis is the -$\log_{10}$ p-value and the dotted line signifies p = 0.05.

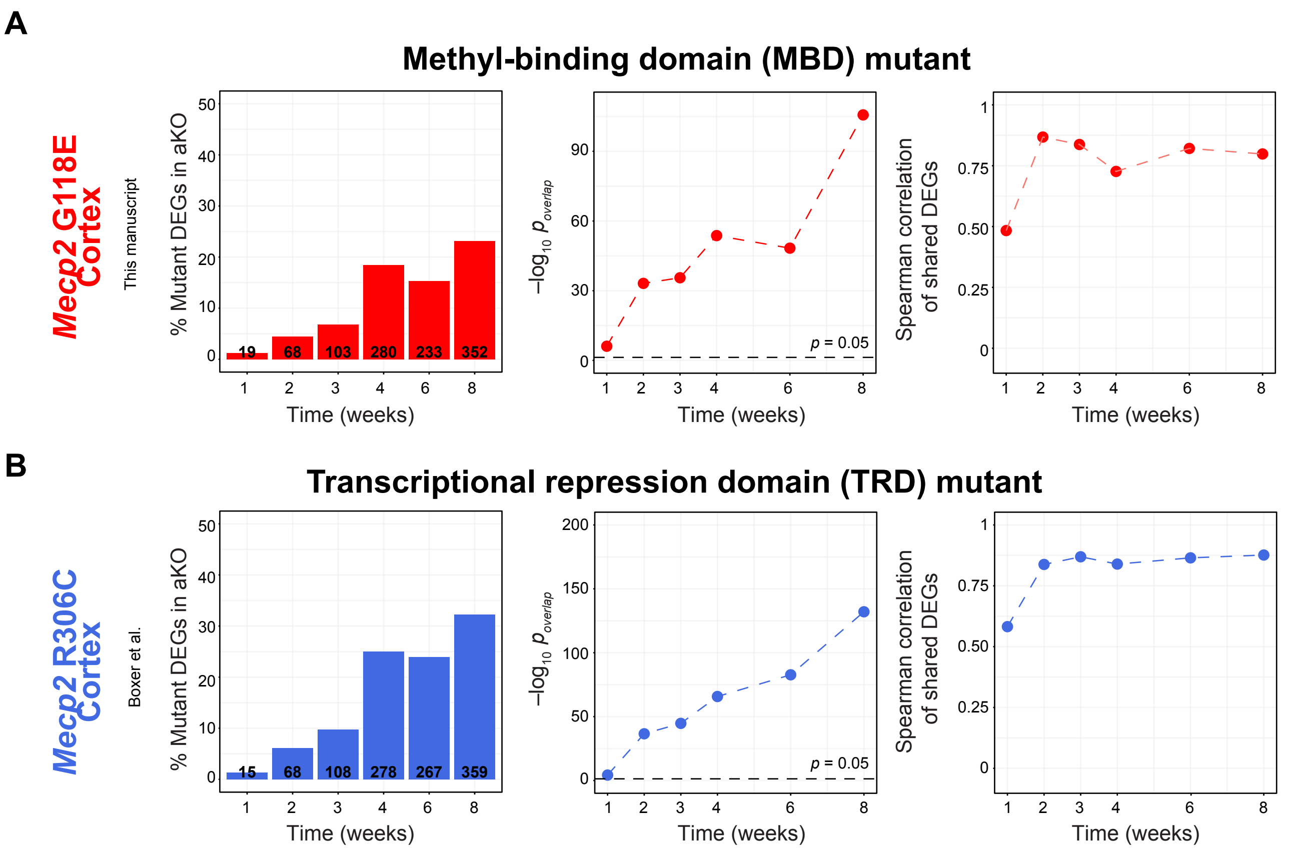

Figure S5. **Related to Figure 3. Transcriptional dysregulation caused by loss of MeCP2 in adulthood is similar to the transcriptional dysregulation caused by mutations in key domains of MeCP2.** Differential gene expression (DEG) from aKO hippocampi (Figure 2) was compared to DEGs identified from transcriptomes collected from the cortices of mouse models harboring patient-relevant point mutations in *Mecp2*. (A) Comparison of DEGs in aKO with a methyl-binding domain (MBD) mutant model, *Mecp2*-G118E. (B) Comparison of DEGs in aKO with a transcriptional repression domain (TRD) mutant model, *Mecp2*-R306C [S1]. For each study, the number and percentage of aKO DEGs present in the point mutant model, the probability of overlap, and the Spearman correlation between shared DEGs was calculated for each time point of aKO samples. The total number of DEGs shared between each comparison is shown in black text on the respective bar. The probability of overlap was computed by Fisher's exact test and the dashed black line indicates p = 0.05.

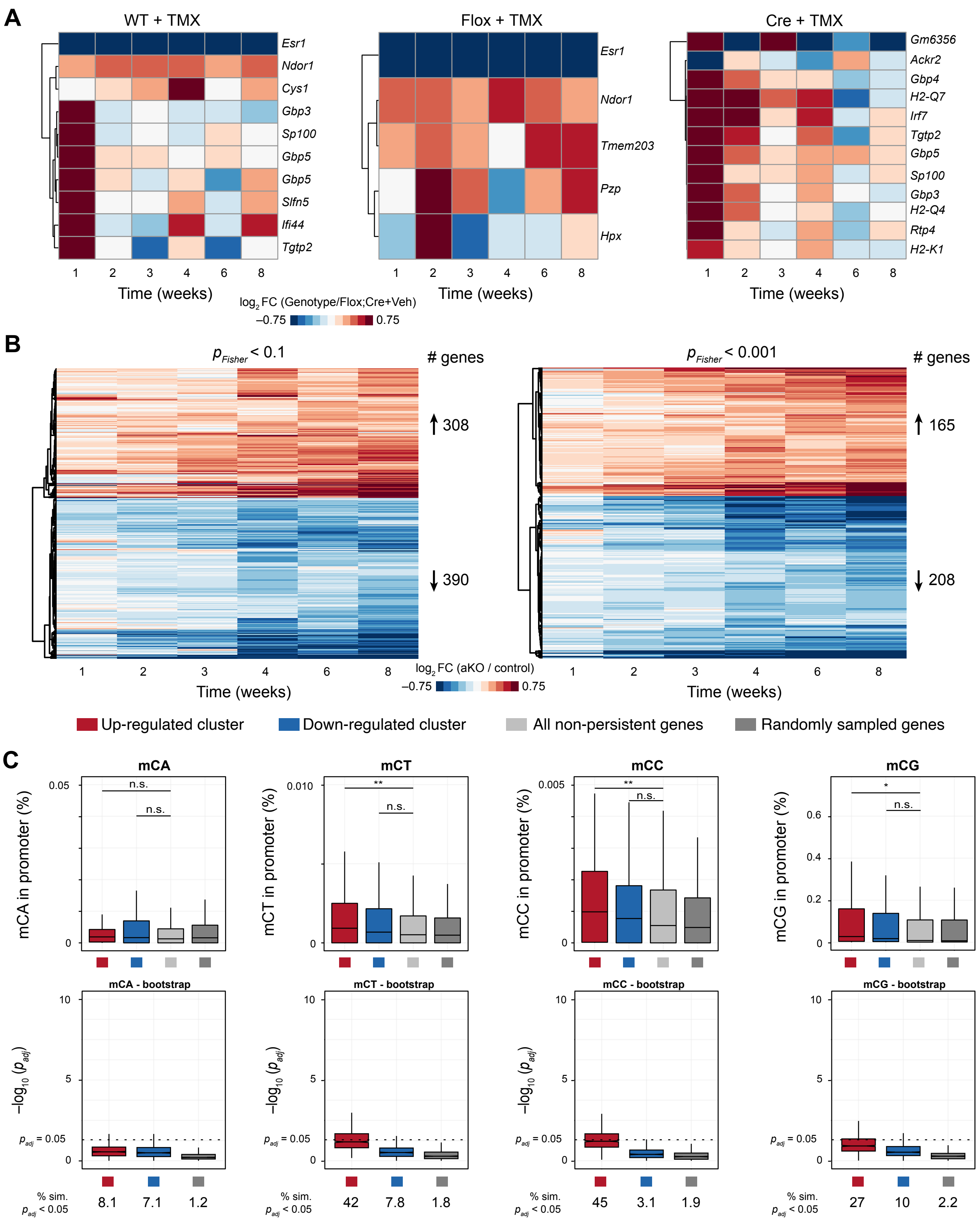

Figure S6. Related to Figure 4. MeCP2-specific persistently dysregulated genes are highly methylated, and the up-regulated persistently dysregulated genes tend to be longer. (A) Heatmap of the genes persistently dysregulated after loss of MeCP2 as determined by a Fisher's aggregate *p*-value < 0.1 (see Methods). Of note, down-regulation of *Esr1* in wild-type and Flox only animals is due to not expressing the

CreERT2 fusion allele. Up-regulation of *Ndor1* in wild-type and Flox only animals is due to not expressing CreER, which is integrated into the *Ndor1* locus. (B) Heatmap of the genes persistently dysregulated after loss of MeCP2 as determined by a Fisher's aggregate p-value < 0.1 and 0.001 and a minimal change of at least 20% at one point during the time course. The number of up- and down-regulated genes is highlighted to the right of the heatmap. (C) Percentage of length-normalized methylated cytosine dinucleotide contexts within the promoter (2 kb upstream of TSS) was tabulated from a previously published methylation study [S2]. Top row displays a boxplot of the percentage of methylation grouped by up-regulated, down-regulated, or non-persistently regulated genes (gray). Dark gray displays a random sample of non-persistently regulated genes of nearly equal sample size as of the persistently dysregulated genes. Bottom row displays the distribution of p_{adj}-values of Kruskal-Walis test followed by Dunn's multiple comparisons comparing up-regulated, down-regulated, or non-persistently dysregulated genes to 1000 bootstrapped simulated subsamples of non-persistently dysregulated genes. The percentage of simulations with $p_{\mathrm{adj}} < 0.05$ are shown below the boxplots.

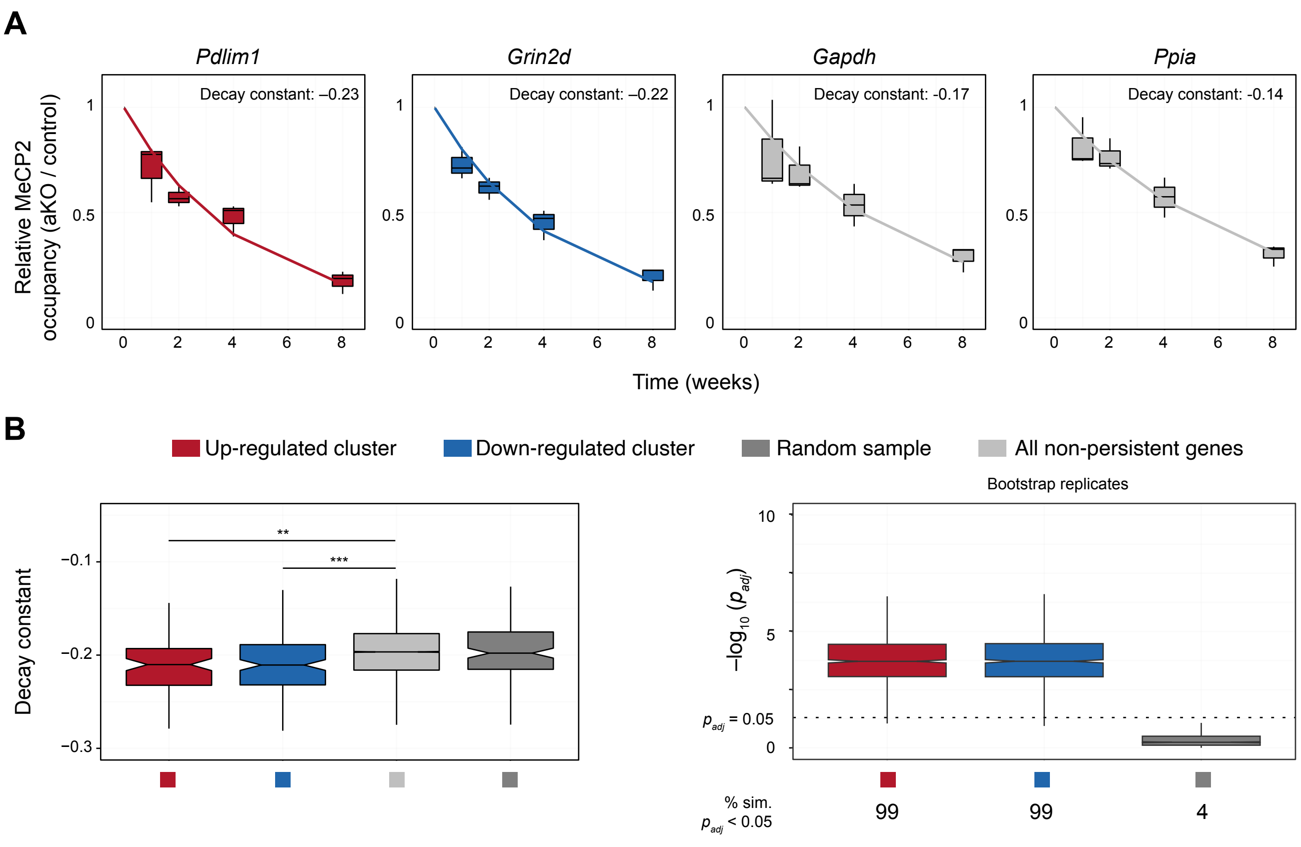

Figure S7. Related to Figure 5. MeCP2 is depleted with a higher decay constant at persistently dysregulated genes. (A) Summarizing MeCP2 depletion at individual loci using an exponential decay fit. Integrated signals of MeCP2 binding over time are shown for four genes: *Pdlim1* (up-regulated), *Grin2d* (down-regulated), and *Gapdh* and *Ppia* (non-dysregulated). The boxplot displays the MeCP2 signal integrated from the promoter and gene body of the aKO condition normalized to the control condition for three biological replicates. The solid line represents an exponential decay fit to the data (see Methods). The extracted decay constant from each curve is shown on the upper right of each graph. (B) Decay constants for every detected gene were tabulated and sorted into up- and down-regulated genes and non-dysregulated genes; (**) $P < 0.01$ and (***) $P < 0.001$. Dark gray displays decay constants from random sample of non-persistently regulated genes of nearly equal sample size as of the persistently dysregulated genes. Bottom row displays the distribution of p_{adj}-values of Kruskal-Walis test followed by Dunn's multiple comparisons comparing up-regulated, down-regulated, or non-persistently dysregulated genes to 1000 bootstrapped simulated subsamples of non-persistently dysregulated genes. The percentage of simulations with $p_{\mathrm{adj}} < 0.05$ are shown below the boxplots.

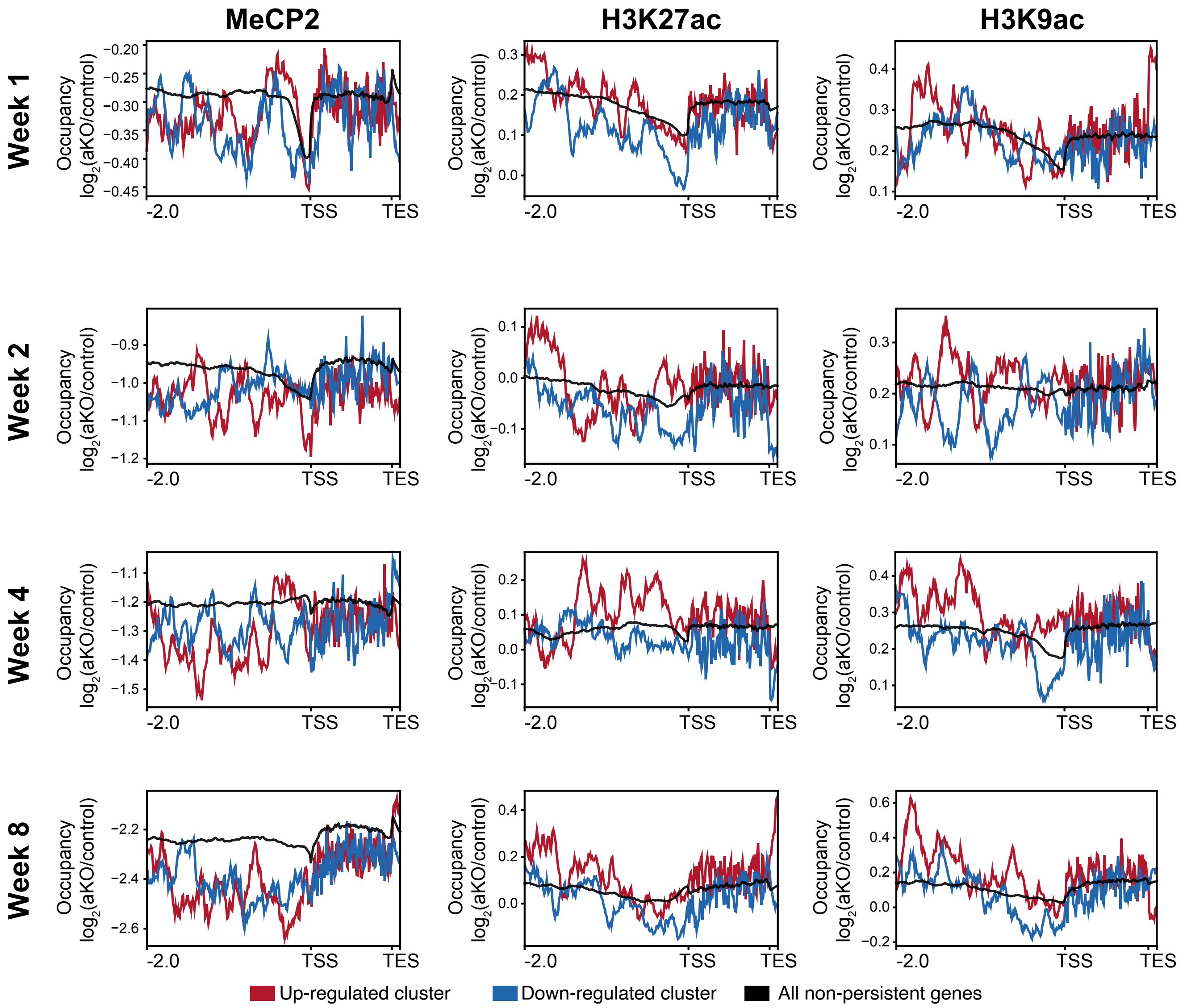

Figure S8. Related to Figure 6. Profile plot view of CUT&RUN data. The $\log_2$ ratio between aKO and control CUT&RUN signal intensity is plot from -2 kb to the transcriptional end site (TES), which is the area of signal that was integrated in the main and supplemental figures. The signal intensities are grouped by gene category and collapsed into an aggregate track, where red is the up-regulated cluster and blue is the down-regulated cluster from Figure 4. Black are the remaining non-persistently dysregulated genes. The area from the transcriptional start site (TSS) to TES is normalized per gene and is referred to as a “meta-gene”. Signals are aggregated from n = 3 profiles per genotype and the $\log_2$ ratio between aKO and control were plotted.

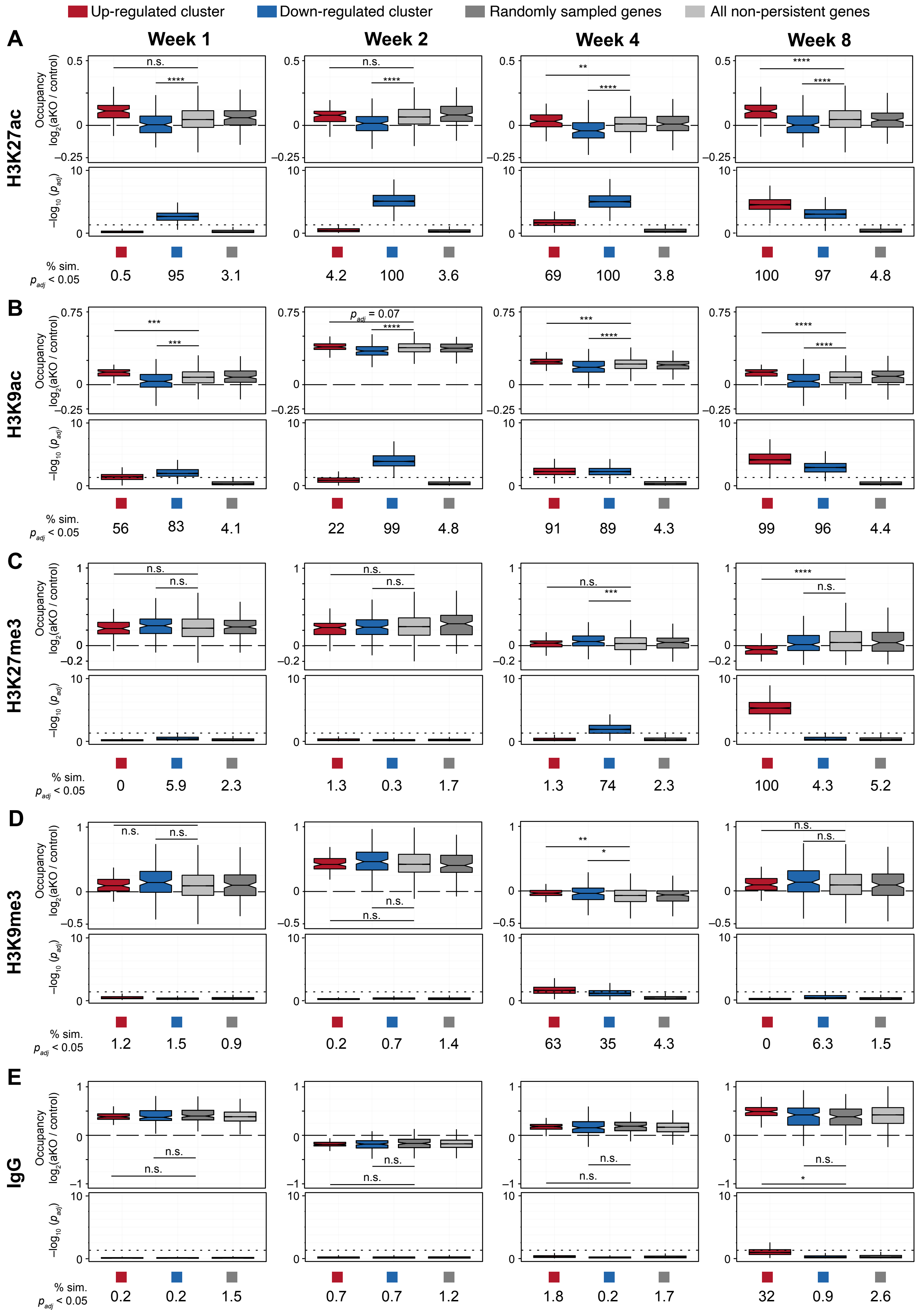

Up-regulated cluster
Down-regulated cluster
Randomly sampled genes
All non-persistent genes
A
B
C
D
E
Week 1
Week 2
Week 4
Week 8
H3K27ac
H3K9ac
H3K27me3
H3K9me3
IgG
Occupancy log2(aKO / control)
−log10 (padj)
% sim. padj < 0.05
n.s.
padj = 0.07
0.5 95 3.1
4.2 100 3.6
69 100 3.8
100 97 4.8
56 83 4.1
22 99 4.8
91 89 4.3
99 96 4.4
0 5.9 2.3
1.3 0.3 1.7
1.3 74 2.3
100 4.3 5.2
1.2 1.5 0.9
0.2 0.7 1.4
63 35 4.3
0 6.3 1.5
0.2 0.2 1.5
0.7 0.7 1.2
1.8 0.2 1.7
32 0.9 2.6

Figure S9. Related to Figure 6. Loss of MeCP2 acutely modulates the occupancy of histone modifications near persistently dysregulated genes. (A-E) Integrated density of CUT&RUN signals for the respective mark shown on the left. For each measured mark, the signal intensity is displayed as the $\log_2$ ratio of aKO to control; genes were then grouped as either persistently up-regulated (red), down-regulated (blue), or the background genome (light gray) and displayed as a boxplot. Differences in the medians of each group was assessed by Kruskal-Wallis test followed by Dunn's multiple comparisons correction. To control for differences in size of groups, we also compared the up- and down-regulated genes (dark gray) to a random subsample of non-persistently dysregulated genes of similar gene number (see Methods); (*) $P < 0.05$, (**) $P < 0.01$, (***) $P < 0.001$, and (****) $P < 0.0001$. Bottom row displays the distribution of p_{adj}-values of Kruskal-Walis test followed by Dunn's multiple comparisons comparing up-regulated, down-regulated, or non-persistently dysregulated genes to 1000 bootstrapped simulated subsamples of non-persistently dysregulated genes. The percentage of simulations with $p_{adj} < 0.05$ are shown below the boxplots. The Z-scored normalized signal for Figure S9 is shown in Figure 6.

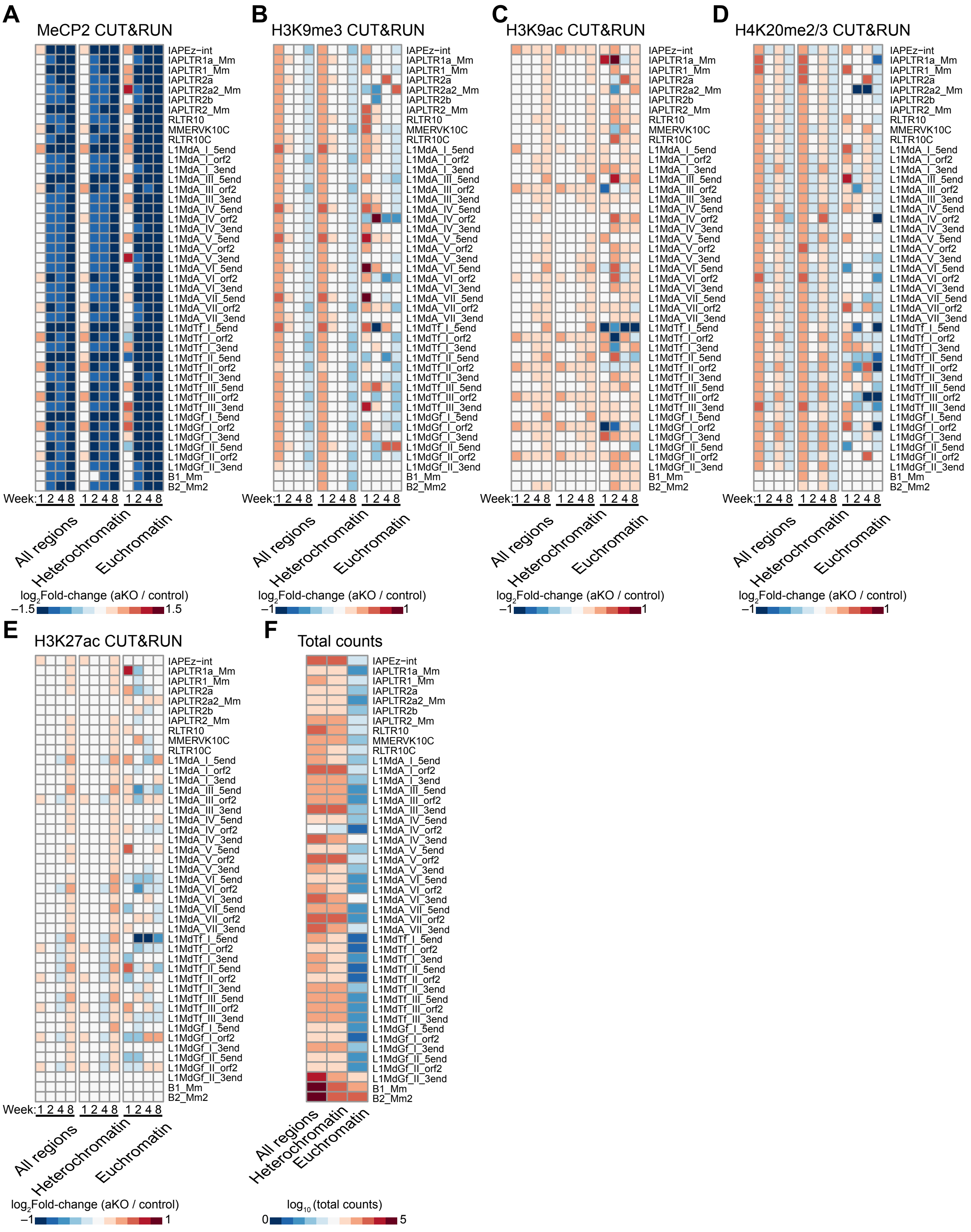

Figure S10. Related to Figure 6. Analysis of epigenetic marker occupancy at repeat regions over time after loss of MeCP2. DNA occupancy of MeCP2 (A), H3K9me3 (B), H3K9ac (C), H4K20me2/3 (D), and H3K27ac (E) was quantified as repeat region elements over time using CUT&RUN. The genomic regions analyzed were further subset by euchromatin (co-localization with H3K27ac peaks) or heterochromatin (co-

localization with H3K9me3), respectively (see Methods). (F) The $\log_{10}$ of total counts from the genomic annotations of a repeat family per genome bin. Rows are individual repeat family type and color represents the signal intensity. Heatmap data and associated Wilcoxon test of aKO versus control are presented in Table S7.

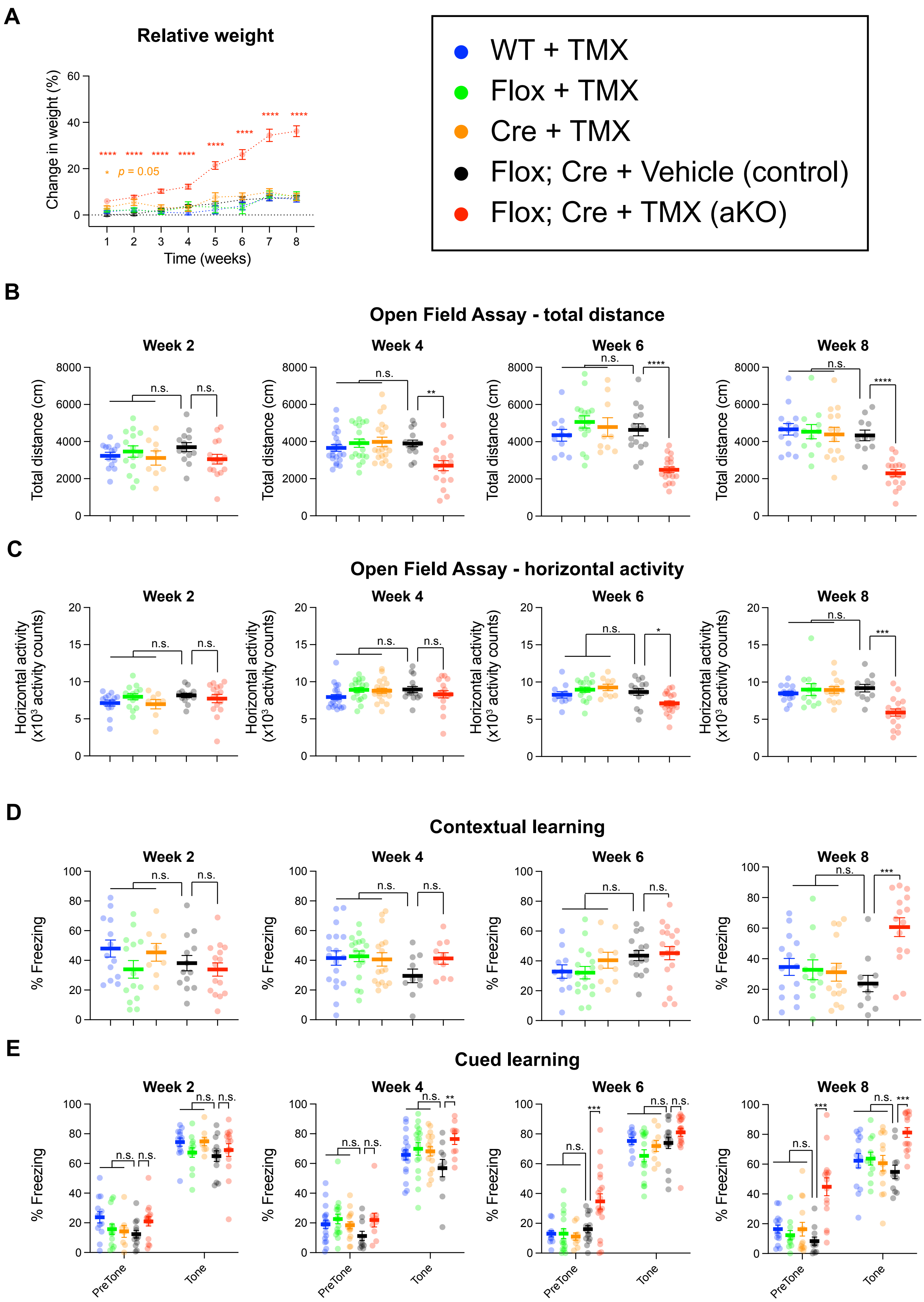
A
Relative weight
Change in weight (%)
Time (weeks)
p = 0.05
WT + TMX
Flox + TMX
Cre + TMX
Flox; Cre + Vehicle (control)
Flox; Cre + TMX (aKO)
B
Open Field Assay - total distance
Week 2
Week 4
Week 6
Week 8
Total distance (cm)
C
Open Field Assay - horizontal activity
Horizontal activity (x10^3 activity counts)
D
Contextual learning
% Freezing
E
Cued learning
PreTone
Tone
n.s.

Figure S11. Related to Figure 7. Physiological and behavioral assessments during adult knockout time course. Mice were treated with vehicle or tamoxifen (TMX) as described in Figure 1. (A) Percent change in weight aKO, control, or treatment control (WT+TMX, Flox+TMX, and Cre+TMX) over time compared to the weight taken just prior to first intraperitoneal injection (n = 11-42). (B,C) Open field assay metrics of total distance traveled (cm) and horizontal activity counts over time (n = 8-22). (D,E) Contextual and cued learning metrics, as assessed by freezing, over time (n = 8-19). Data were analyzed by two-way ANOVA (A) or one-way ANOVA (B,C) and Dunnett's post-hoc multiple comparisons, with (*) $P < 0.05$, (**) $P < 0.01$, (***) $P < 0.001$, and (****) $P < 0.0001$.

SUPPLEMENTAL REFERENCES

S1. Boxer, L.D., Renthal, W., Greben, A.W., Whitwam, T., Silberfeld, A., Stroud, H., Li, E., Yang, M.G., Kinde, B., Griffith, E.C., et al. (2020). MeCP2 Represses the Rate of Transcriptional Initiation of Highly Methylated Long Genes. Mol Cell *77*, 294-309 e9. https://doi.org/10.1016/j.molcel.2019.10.032.

S2. Reizel, Y., Sabag, O., Skversky, Y., Spiro, A., Steinberg, B., Bernstein, D., Wang, A., Kieckhaefer, J., Li, C., Pikarsky, E., et al. (2018). Postnatal DNA demethylation and its role in tissue maturation. Nat Commun *9*, 2040. https://doi.org/10.1038/s41467-018-04456-6.
